# Interaction between Ca_V_2.1 and Junctophilin3/4 depends on the II-III loop of Ca_V_2.1 and on the α-helical region of Junctophilin3/4

**DOI:** 10.1016/j.jbc.2025.108424

**Published:** 2025-03-19

**Authors:** Stefano Perni, Alexander Polster, Kurt G. Beam

**Affiliations:** Department of Physiology and Biophysics, University of Colorado, Aurora, Colorado, USA

**Keywords:** Ca_V_2.1, ER-PM junctions, junctophilin, calcium signaling, protein–protein interaction

## Abstract

Neuronal junctophilins (JPH3 and JPH4) form junctions between the endoplasmic reticulum (ER) and plasma membrane (PM) through their C-terminal transmembrane (TM) domain, which is embedded in the ER membrane, and N-terminal domain, which binds to the PM. JPHs also recruit and slow the inactivation of the voltage-gated Ca^2+^ channel Ca_V_2.1. Here, we identified the domains responsible for Ca_V_2.1–JPH interactions by co-expressing the isolated GFP-tagged Ca_V_2.1 cytoplasmic domains with mCherry-tagged JPH3/4 in tsA201 cells. Among the Ca_V_2.1 domains, only the II-III loop colocalized with JPH3 and JPH4 as well as with the TM-truncated JPH3-ΔTM and JPH4-ΔTM constructs, which cannot form ER–PM junctions. Further fragmentation of the II-III loop showed that both JPH-ΔTM constructs colocalized with the proximal half of the loop containing the synprint domain, known to bind presynaptic proteins, but only JPH4-ΔTM colocalized with the distal half and only JPH4 slowed the inactivation of a Ca_V_2.1 construct lacking most of the synprint region. JPH colocalization with the II-III loop persisted when JPH divergent and TM domains were deleted but was lost when the α-helical domain was also removed. Swapping the α-helical domains between JPH3 and JPH4 led to a corresponding exchange in their ability to interact with the II-III loop distal segment. Thus, the α-helical domain appears necessary for JPH binding to the synprint-containing II-III loop half and for the differential binding of JPH3 and JPH4 to the loop distal half. Furthermore, the binding of JPH α-helical domain to the Ca_V_2.1 II-III loop is essential for slowing Ca_V_2.1 inactivation.

Voltage-gated calcium channels are critical for the function of excitable cells. These channels are composed of multiple subunits, including an α-subunit, which governs the major properties of the channel. Based on sequence, α subunits fall into three groups: Ca_V_1 (with four subtypes), Ca_V_2 (three subtypes), and Ca_V_3 (three subtypes). All Ca_V_ α subunits share a similar architecture consisting of four transmembrane (TM) domains (designated by Roman numerals), each formed by six TM helices. The four domains are connected by intracellular loops, designated I-II, II-III, and III-IV. The N- and C-terminals are also intracellular, leaving only a small fraction of the channel exposed to the extracellular environment. The TM helices that form the four domains contain the channel's voltage-sensing and pore-forming domains; the intracellular regions are available for interactions with auxiliary subunits and regulatory proteins that modulate the properties and trafficking of the channels.

One important role for Ca_V_2.1 is in nerve terminals, where it triggers neurotransmitter release in response to an action potential (1, 2). To function properly, this process requires close proximity between Ca_V_2.1 and the proteins responsible for the docking and fusion of the synaptic vesicles with the presynaptic plasma membrane (PM). This close spatial association is facilitated by a direct interaction between Ca_V_2.1 and members of the snap receptor proteins such as syntaxins and synaptotagmin I in the nerve terminals. This interaction is mediated by the so-called synaptic protein interaction (synprint) domain located in the II-III loop of Ca_V_2.1 (3, 4, 5).

In addition to being present in nerve terminals, Ca_V_2.1 is also present in other neuronal regions. Thus, the application of freeze replica immunolabeling has revealed that nonsynaptic Ca_V_2.1 channels in cerebellar Purkinje neurons are arrayed in two distinct patterns: a population of diffusely distributed channels whose density increases towards the distal dendrites and a population of channels densely packed in submicrometer puncta (200–400 nm diameter) which are mostly present in the soma or proximal dendrites; the latter population is closely associated in the PM with BK and SK calcium-activated potassium channels (6). It seems likely that the diffusely distributed Ca_V_2.1 channels support dendritic calcium spikes, for which Ca_V_2.1 is known to be important (7), and that the Ca_V_2.1 in punctate clusters participates in the generation of the slow after-hyperpolarization (sAHP) which follows complex spikes in Purkinje neurons. Based on pharmacological analysis (8, 9), the sAHP in Purkinje neurons depends upon a tripartite complex consisting of Ca_V_2.1 and SK calcium-activated potassium channels in the PM and ryanodine receptors (RyRs) in the underlying endoplasmic reticulum (ER) membrane (which would not have been present in the preparations analyzed by freeze replica immunolabeling). Specifically, this pharmacological analysis suggests that Ca^2+^ entry *via* Ca_V_2.1 triggers Ca^2+^ release from the ER *via* ryanodine receptors, which activates SK channels and results in hyperpolarization. An analogous tripartite complex between L-type Ca_V_s (Ca_V_1.2 and Ca_V_1.3), type-2 ryanodine receptors (RyR2), and SK4 Ca^2+^-activated potassium channels generates the sAHP in hippocampal pyramidal neurons (10). Moreover, recent work has demonstrated that in proximal and medial dendrites of hippocampal neurons, there are junctions between the PM and ER, which contain JPH3 and JPH4 (11). The functional interaction between Ca_V_s and SKs in the PM and RyRs in the ER requires subcellular structures, termed ER–PM junctions, at which these two membranous compartments are closely apposed. The formation and stabilization of ER–PM junctions is mediated by specialized proteins, including junctophilins (JPHs), that can, by themselves or in association with other proteins, form a bridge between the PM and ER membranes (12). The four JPHs isoforms share a similar architecture: two sets of N-terminal membrane occupation and recognition nexus (MORN) domains, which are separated by a "Joining" domain, an "α-helical" domain, a "divergent" domain, and a short C-terminal domain embedded in the ER membrane. The JPHs bind to the internal leaflet of the PM, possibly through their MORN domains (13). As a result, JPHs form and stabilize ER–PM junctions. Of the four JPH isoforms, two (JPH1 and JPH2) are expressed in muscle and two (JPH3 and JPH4) are broadly expressed in the brain (14). Knockout of both neuronal JPHs results in a loss of the sAHP and impaired long-term potentiation in the hippocampus at the cellular level and memory defects and aberrant hindlimb reflexes at the whole animal level (15). The JPH3/JPH4 double KO also results in a loss of the sAHP and long-term depression in cerebellar Purkinje neurons (8).

Because the JPHs can recruit a variety of different calcium-signaling proteins to ER–PM junctions, it seems likely that there are binding interactions between the JPHs and one or more of these signaling proteins. This has been demonstrated in the arguably best-characterized ER–PM junctions, the triad junctions. Specifically, the skeletal muscle L-type Ca^2+^ channel (Ca_V_1.1) co-immunoprecipitates with both JPH1 and JPH2 (16), and pull-down assays of GST-tagged peptides, and mutagenesis of full-length Ca_V_1.1, indicate that this interaction depends on a 10 to 15 residue stretch of amino acids, which lie downstream from the IQ domain in Ca_V_1.1 C-terminal (17). Within this region, the motif IFFRxGGLFG is also present in the Ca_V_1.2 C-terminus (17, 18) and is partially conserved in Ca_V_1.3 (19). This motif may also be responsible for the recruitment of Ca_V_1.1 and Ca_V_1.2 to ER–PM junctions induced by either JPH3 or JHP4 in HEK293-derived cells (20, 21). However, this motif does not exist in Ca_V_2.1 and thus cannot be involved in the recruitment of this channel to junctions induced by JPH3 and JPH4 nor for the ability of both neuronal JPHs to greatly slow the inactivation of current *via* Ca_V_2.1, with no effect on the channel’s steady-state activation (21). This slowing of inactivation would be expected to increase Ca^2+^ entry at ER–PM junctions containing Ca_V_2.1 together with JPH3 and/or JPH4.

Here, we have used expression in tsA201 cells of Ca_V_2.1 and of the neuronal JPHs to identify structural determinants important for both recruitment to ER–PM junctions (6, 11) and for slowing of inactivation (21). Recruitment was assessed by the extent to which fluorescently tagged constructs colocalized with one another and the slowing of inactivation by whole cell measurements of Ca^2+^ currents. In the case of inactivation, the experiments addressed why JPH4 causes a greater reduction in the inactivation rate than JPH3 (21). We found that, unlike the L-type channels, recruitment to junctions appears to depend on the binding of JPH3 and JPH4 to the II-III loop of Ca_V_2.1. JPH3 and JPH4 constructs truncated after the α-helical domain retained the ability to interact with the II-III loop, whereas those in which most, or all, of the α-helical domain was additionally deleted, lost the ability to interact with the loop. The determinants for ER–PM junction recruitment within the loop differed for the two JPHs: both colocalized with the proximal half of the II-III loop that contains the synprint domain, whereas JPH4 additionally colocalized with the distal region of the loop that is C-terminal to the synprint domain. This behavior was inverted when the α-helical domains were exchanged between JPH3 and JPH4. However, the greater slowing of inactivation by JPH4 than by JPH3 was not inverted by exchanging the α-helical domains, and this differential effect on inactivation must, therefore, be a consequence of JPH regions outside the α-helical domain.

## Results

### JPH3 and JPH4 bind to the II-III loop of Ca_V_2.1

**Figure 1:**
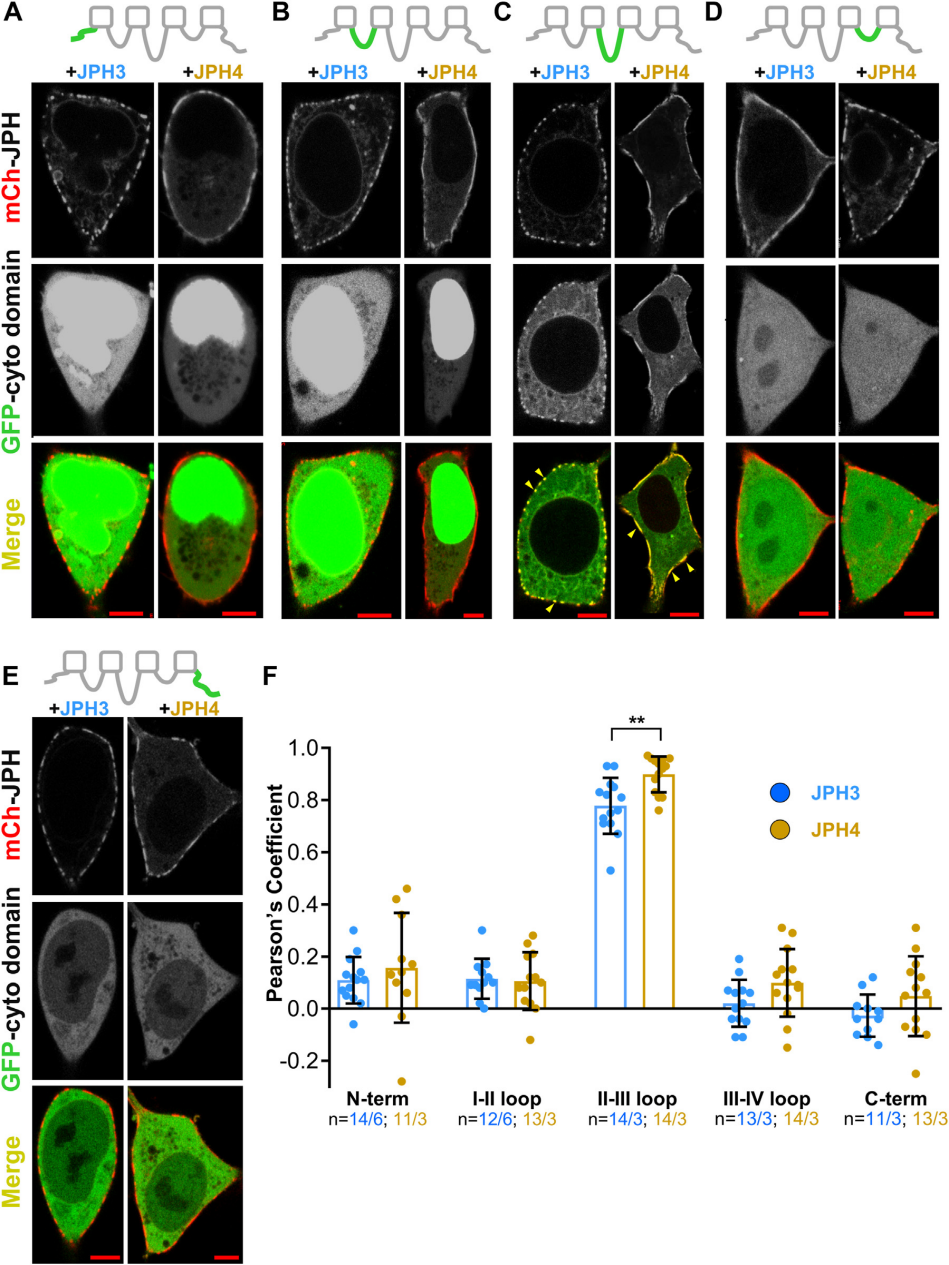
**JPH3 and JPH4 interact with the Ca_V_2.1 II-III loop.** Mid-level confocal scans of tsA201 cells transfected with mCherry-tagged JPH3 or JPH4 together with the isolated, GFP-tagged Ca_V_2.1 cytoplasmic domains: N-terminus (*A*), I-II loop (*B*), II-III loop (*C*), III-IV loop (*D*), and C-terminus (*E*). For each combination of constructs, the three panels arrayed vertically illustrate the distribution of the junctophilin (*red*, topmost), cytoplasmic domain (*green*, center), and the overlay of these two images (*bottom*-most). At the cell periphery, the Ca_V_2.1 II-III loop colocalized with both JPH3 and JPH4, as revealed in the overlaid images by the presence of *yellow* puncta, some of which are indicated by arrowheads (*C*). There appeared to be no colocalization of any other Ca_V_2.1 cytoplasmic domains with either JPH3 or JPH4. Bars represent 5 μm. *F*, Pearson's colocalization coefficients calculated from confocal sections at the *bottom* surface of the cell. Data are reported as values from individual cells (*circles*), with the mean indicated by the height of the superimposed rectangles and ± SD by the horizontal *black* lines. For each construct combination, the numbers indicate total number of analyzed cells/number of separate transfected dishes. The colocalization between the II-III loop and JPH4 appeared to be somewhat greater than that between the loop and JPH3 (∗∗*p* = 0.0019, *t* test with Welch's correction).

We previously showed that the expression of fluorescently tagged neuronal JPHs in tsA201 cells induces the formation of ER–PM junctions, which were evident in thin-section electron micrographs and which resulted in the presence of small (∼μm or less) puncta of fluorescence at the cell surface (21). Additionally, we found that Ca_V_2.1 was recruited to these junctions (21). To identify cytoplasmic domains of Ca_V_2.1 important for its recruitment into junctions, we have now constructed complementary DNAs encoding GFP fused to Ca_V_2.1 fragments corresponding to the N-terminal, I-II loop, II-III loop, III-IV loop, and C-terminal and expressed each of them in tsA201 cells together with JPH3 or JPH4 tagged with mCherry. Figure 1 shows representative images of midlevel confocal scans of such cells with each of the Ca_V_2.1 cytoplasmic domains indicated in green and JPH3 or JPH4 indicated in red. For all the construct combinations, both JPHs were concentrated in discrete foci at the cell's periphery, presumably representing ER–PM junctions. By contrast, the subcellular distribution differed between the Ca_V_2.1 cytoplasmic domains. Specifically, the N-terminal and I-II loop constructs were present at the highest concentration in the nucleus but also distributed throughout the cytoplasm; they also appeared to be excluded from the ER lumen as indicated by the small dark, roughly circular structures (Fig. 1, *A* and *B*). The II-III loop construct appeared to be partly cytoplasmic and partly ER-associated, with this ER association indicated by bright, roughly circular structures with dark interiors (Fig. 1*C*). The III-IV loop was present at similar levels in the nucleus and cytoplasm and excluded from the ER lumen (Fig. 1*D*), and the C-terminal was predominantly cytoplasmic but also present in the nucleus (Fig. 1*E*). Of the five cytoplasmic constructs, only the II-III loop was arrayed at the cell periphery in a pattern overlapping that of both JPH3 and JPH4 (Fig. 1*C*). To quantify colocalization, we calculated Pearson's coefficients for colocalization between the Ca_V_2.1 cytoplasmic domains and JPHs from confocal scans acquired at the cells' bottom surface. Based on Pearson's coefficients, the colocalization between the II-III loop and JPH4 was slightly, but significantly (*p* = 0.0019), greater than that between the loop and JPH3 (Fig. 1*F*).

One possible explanation for why the GFP-tagged cytoplasmic domains of Ca_V_2.1, except for the II-III loop, did not colocalize with JPH3 or JPH4 is that the observed green fluorescence arose from unattached GFP. Thus, we also tested Ca_V_2.1 cytoplasmic domains which were tagged at their amino terminals with GFP-(Ca_V_1.2 I-II loop), which associates with the PM because the I-II loop of Ca_V_1.2 contains a polybasic sequence that interacts with membrane phosphoinositides (22). We tested these constructs by co-expressing them with JPH3 (Fig. S1*A*) and found that all of them produced a prominent, surface-associated fluorescence but that only the fluorescence for the II-III loop construct colocalized with JPH3 (Fig. S1, *B*–*G*). However, we did not pursue the use of these constructs further because we found that the association of GFP-(Ca_V_1.2 I-II loop)-(Ca_V_2.1 II-III loop) with both the PM and ER resulted in the induction of ER–PM junctions when expressed alone, as indicated by its segmented distribution at the cell surface (Fig. S1*H*). This pattern was not observed for the construct GFP-(Ca_V_2.1 I-II loop) which appeared to associate only with the ER (Fig. S1*I*). In any event, the data from the Ca_V_2.1 cytoplasmic domains attached directly to GFP (Fig, 1) or attached to the GFP-tagged I-II loop of Ca_V_1.2 (Fig. S1) indicate that the II-III loop is a key determinant for the recruitment of Ca_V_2.1 by the neuronal JPHs.

### The ER-anchoring segment of the neuronal JPHs is not required for their association with the Ca_V_2.1 II-III loop

**Figure 2:**
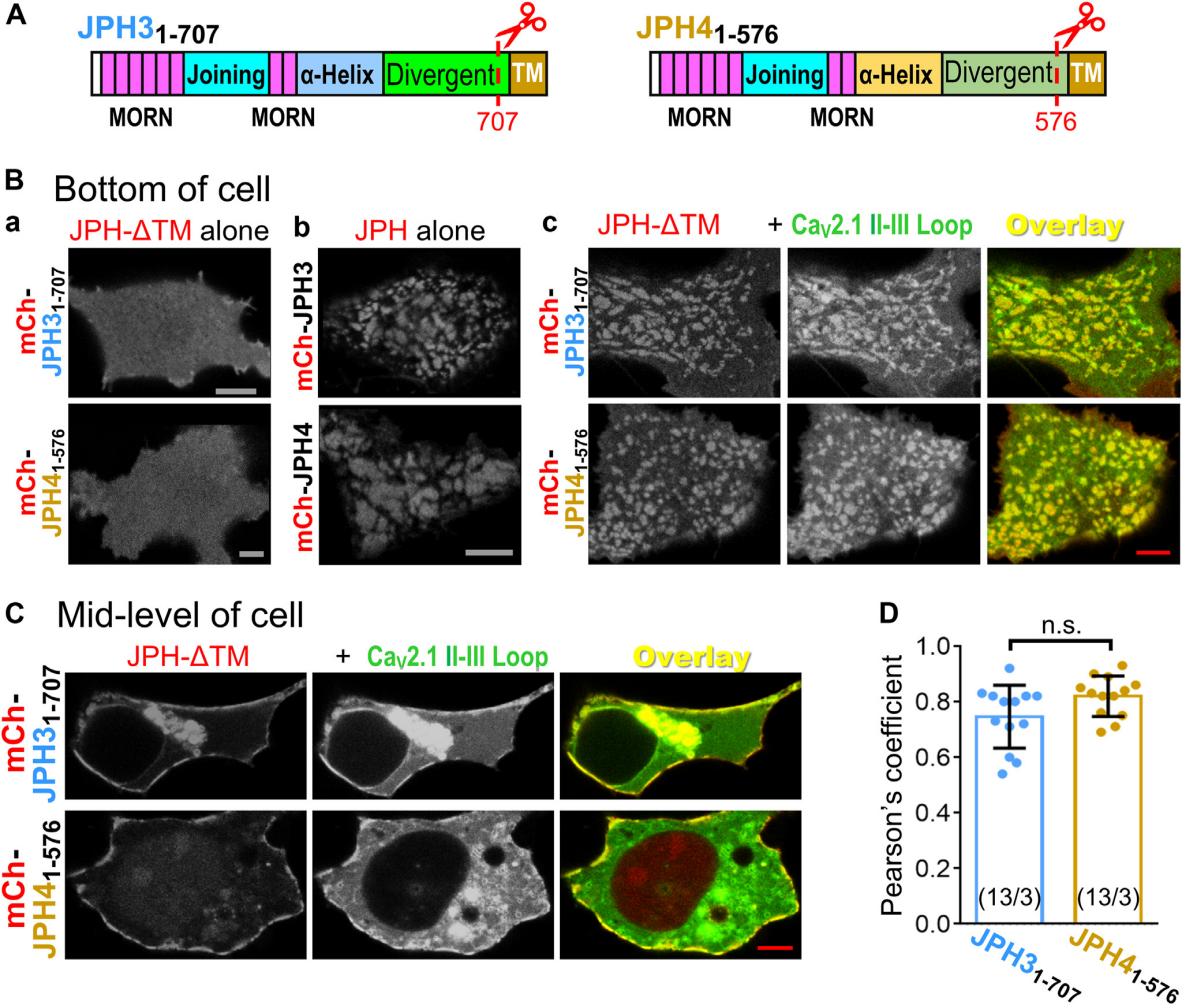
**JPH3 and JPH4 lacking the ER transmembrane segment retain the ability to interact with the Ca_V_2.1 II-III loop, and this interaction appears to rescue junction formation.***A*, schematic representation of JPH-ΔTM constructs, JPH3_1-707_ and JPH4_1-576_, both of which lack the TM domain that anchors them to the ER but retain the N-terminal regions responsible for association with the plasma membrane. *B*, bottom surface optical sections of tsA201 cells transfected with mCherry-JPH3_1-707_ or mCherry-JPH4_1-576_ alone (*a*), full-length mCherry-JPHs alone (*b*), and mCherry-JPH3_1-707_ or mCherry-JPH4_1-576_ in combination with the GFP-Ca_V_2.1 II-III loop (*c*). In the absence of the II-III loop, the truncated junctophilins were distributed evenly on the plasma membrane (*a*). The interaction between the II-III loop, which is associated with the ER, and the JPH-ΔTMs, which are associated with the plasma membrane, was strong enough to rescue ER-PM junction formation, which caused redistribution of the truncated junctophilins into a punctate pattern (*c*) resembling the one observed for full-length junctophilins (*b*). *C*, mid-level confocal scans of tsA201 cells transfected with mCherry-JPH3_1-707_ or mCherry-JPH4_1-576_ in combination with the GFP-Ca_V_2.1 II-III loop. Bars represent 5 μm in (*B*) and (C). *D*, Pearson's colocalization coefficients for the specified combinations of II-III loop and junctophilin constructs were calculated from optical sections of the *bottom* of the cell. Data are reported as values from individual cells (*circles*), with the mean indicated by the height of the superimposed rectangles, and ± SD by the horizontal *black* lines (*p* = 0.0613, *t* test with Welch's correction). Numbers in parentheses represent total number of analyzed cells/separate transfected dishes.

As described above, the Ca_V_2.1 II-III loop appears to be at least partly associated with the ER when expressed in tsA201 cells. Accordingly, one possible reason for its colocalization with JPH3 and JPH4 could simply be that the ER-spanning segment of these JPHs is localized in the same ER compartment with which the loop was associated. To address this possibility, we transfected tsA201 cells with the II-III loop construct together with JPHs truncated just prior to the C-terminal, ER-spanning segment (mCherry-JPH3_1:707_ and mCherry-JPH4_1:576_, Figure 2*A*). Confocal scans near the bottom surface of tsA201 cells transfected with only mCherry-JPH3_1:707_ or mCherry-JPH4_1:576_ (Fig. 2*Ba*) revealed that these truncated JPHs are fairly evenly dispersed at the cell surface, rather than localized in the discrete foci that are seen in cells transfected only with fluorescently tagged, full-length JPH3 or JPH4 (Fig. 2*Bb*). This dispersed distribution is likely because the truncated constructs cannot induce ER–PM junctions but retain the ability to bind to the internal leaflet of the PM *via* their intact N-terminal regions. Importantly, the truncated JPH3 and JPH4 constructs retained the ability to colocalize with the GFP-tagged II-III loop, as indicated by the overlapping foci of red and green fluorescence in both bottom surface (Fig. 2*Bc*) and midlevel (Fig. 2*C*) confocal scans. Strikingly, the co-expression of the II-III loop caused a redistribution of JPH3_1:707_ and JPH4_1:576_ from dispersed back to a punctate pattern (Fig. 2, *Bc* and *C*) like that observed for full-length JPHs. As described earlier, when the II-III loop was expressed alone, it showed a reticular distribution without any evident association with the PM (Fig. S1*I*). Thus, it appears more likely that the redistribution of the truncated JPHs results from an interaction between the II-III loop, associated with the ER, and the truncated JPHs bound to the PM, and that this interaction reconstituted ER–PM junctions. The presence of these reconstituted ER–PM junctions allowed us to quantify the colocalization of Ca_V_2.1 II-III loop and JPH3_1:707_ and JPH4_1:576_ from bottom-surface optical sections, which reduces the interference from fluorescence in the internal ER. The Pearson's colocalization coefficients calculated in this way (Fig. 2*D*) were similar to those for the Ca_V_2.1 II-III loop and full-length JPH3 and JPH4, although the slightly higher colocalization values for JPH4_1:576_ than JPH3_1:707_ are not statistically significant. Thus, the colocalization between the II-III loop and JPHs does not require the ER-anchoring segment of the JPHs. Moreover, the association between the isolated ER-bound II-III loop and JPH3_1:707_ or JPH4_1:576_ reconstituted ER–PM junctions even in the absence of the JPHs’ C-terminal TM domains.

### JPH4 associates with a more extensive region of the Ca_V_2.1 II-III loop than JPH3

**Figure 3:**
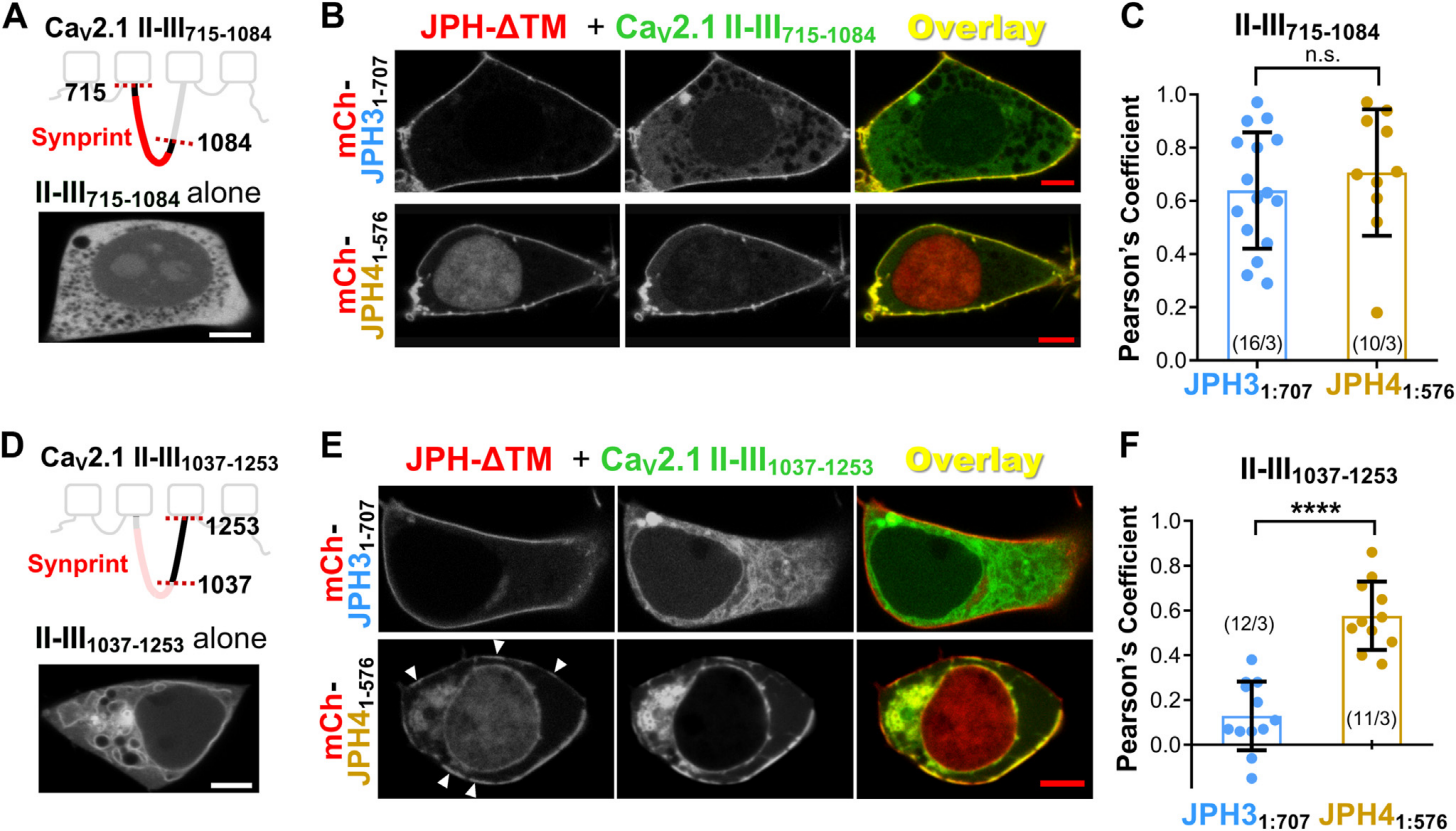
**JPH3-ΔTM and JPH4-ΔTM both interact with the synprint-containing, N-terminal half of the Ca_V_2.1 II-III loop.** Only JPH4-ΔTM interacts with the C-terminal half of the II-III loop. *A*, *top*: schematic representation of Ca_V_2.1 II-III_715-1084_ with the synprint domain indicated in *red*. *Bottom*: midlevel image of a tsA201 cell transfected only with GFP-tagged II-III_715-1084_. *B*, midlevel optical sections of tsA201 cells expressing mCherry-JPH3_1-707_ or mCherry-JPH4_1-576_ together with GFP-Ca_V_2.1 II-III_715-1084_. Co-expression with either JPH3_1-707_ or JPH4_1-576_ caused Ca_V_2.1 II-III_715-1084_ to become concentrated at the cell periphery, where it colocalized with the JPH-ΔTM constructs. *C*, Pearson's coefficients calculated from midlevel optical scans. (n.s. *p* = 0.478, *t* test with Welch's correction). *D*, *top*: schematic representation of Ca_V_2.1 II-III_1037-1253_. *Bottom*: midlevel image of a tsA201 cell transfected only with GFP-tagged II-III_1037-1253_. *E*, midlevel optical sections of tsA201 cells expressing mCherry-JPH3_1-707_ or mCherry-JPH4_1-576_ together with GFP-II-III_1037-1253_. Ca_V_2.1 II-III_715-1084_ did not colocalize with JPH3_1-707_ but did colocalize with JPH4_1-576_. *F*, Pearson's coefficients calculated from bottom surface scans (∗∗∗∗*p* < 0.0001, *t* test with Welch's correction). In (*C*) and (*F*), *circles* indicate values for individual cells, with the mean ± SD indicated by the superimposed rectangle and horizontal *black* lines, respectively; numbers in parentheses indicate the total number of analyzed cells/number of separate transfected dishes. Bars represents 5 μm in (*A*), (*B*), (*D*), and (*E*).

To narrow the region of the Ca_V_2.1 II-III loop responsible for its association with the JPHs, we created two expression plasmids encoding GFP fused either to a proximal loop region (Ca_V_2.1 II-III_715:1084_), which contains the synprint domain (4) or to a distal loop region (Ca_V_2.1 II-III_1037:1253_). When Ca_V_2.1 II-III_715:1084_ was expressed alone, it was diffusely distributed in the cytoplasm (Fig. 3*A*). The additional expression of either JPH3_1:707_ or JPH4_1:576_ caused the recruitment of Ca_V_2.1 II-III_715:1084_ to the periphery of the cell and thus the colocalization with these JPH constructs (Fig. 3*B*). Comparison of the Pearson's coefficients (Fig. 3*C*) indicates that the colocalization of Ca_V_2.1 II-III_715:1084_ with JPH3_1:707_ did not differ significantly from its colocalization with JPH4_1:576_. When Ca_V_2.1 II-III_1037:1253_ was expressed alone (Fig. 3*D*), it appeared to be both diffusely distributed in the cytoplasm and associated with the ER, a pattern similar to that of the full-length II-III loop. Thus, it seems that a region within the distal segment of the loop is responsible for causing the full-length II-III loop to associate with the ER. Unlike Ca_V_2.1 II-III_715:1084_, which was recruited to the surface by both JPH3_1:707_ and JPH4_1:576_, Ca_V_2.1 II-III_1037:1253_ was recruited to the surface only by JPH4_1:576_, a difference which was also apparent in the Pearson's coefficients (Fig. 3*F*). Although we did not attempt to identify specific residues within Ca_V_2.1 II-III_715:1084_ that are critical for its association with JPH3 and JPH4, we did test the subfragments II-III_715-909_ and II-III_900-1084_. Neither of these colocalized with JPH3_1:707_ or JPH4_1:576_ (Fig. S2). Thus, the association of the Ca_V_2.1 II-III loop with the JPHs involves distinct regions. The proximal segment (Ca_V_2.1 II-III_715:1084_) requires an intact synprint domain for colocalization with both JPH3 and JPH4, whereas the distal segment (Ca_V_2.1 II-III_1037:1253_) is recruited to the surface exclusively by JPH4, demonstrating a difference in specificity.

### JPH4, but not JPH3, causes junctional recruitment and slows the inactivation of a Ca_V_2.1 variant that lacks most of the synprint domain

**Figure 4:**
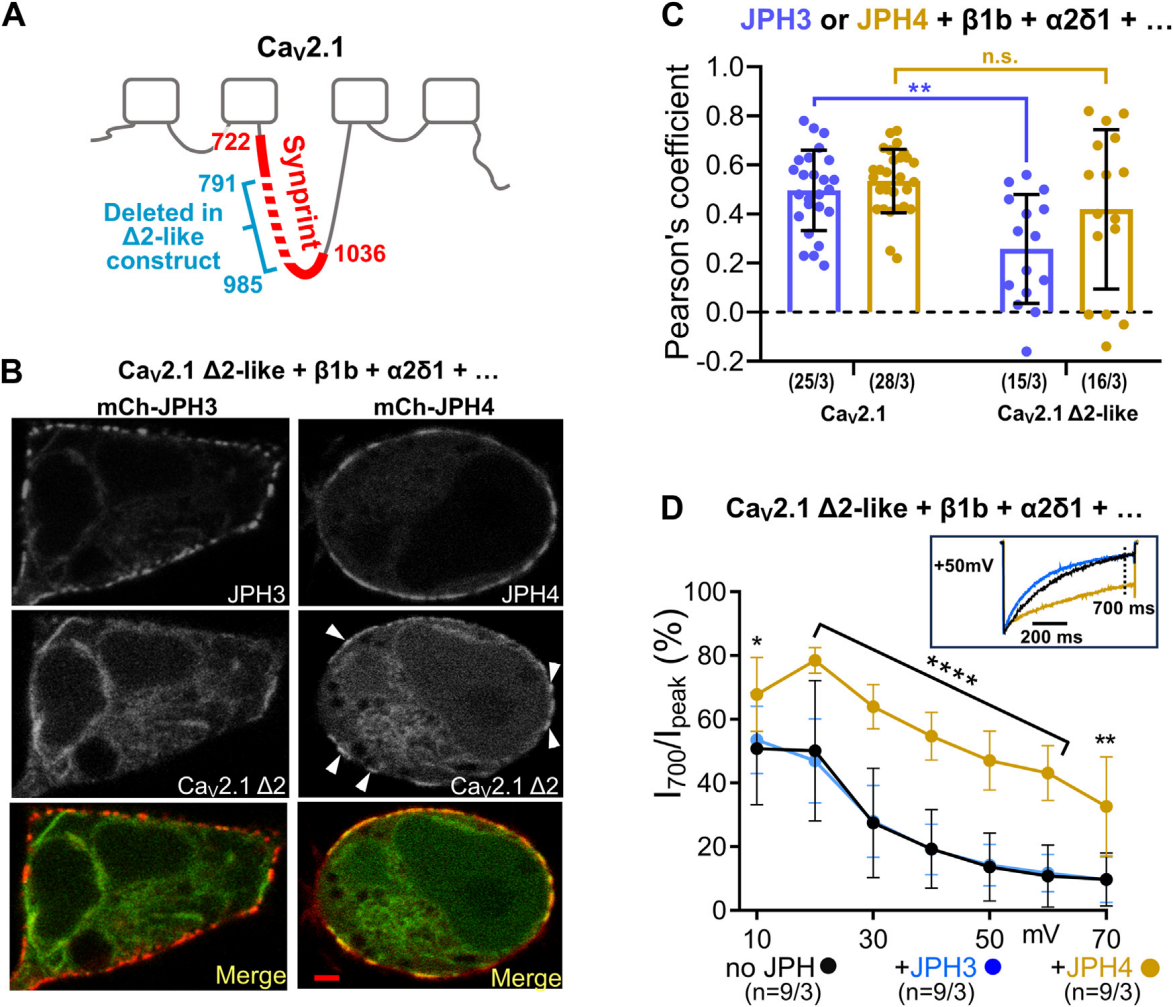
**Recruitment to ER-PM junctions and slowing of inactivation of the Ca_V_2.1 Δ2-like variant, which lacks most of the synprint domain, are mediated by JPH4 but not by JPH3.***A*, schematic representation of rabbit Ca_V_2.1 showing the synprint domain (*red*), with its first and last residues (4) indicated. The *cyan* bracket and numbers indicate the residues deleted to obtain the Δ2-like rabbit variant, based on the rat Δ2 splice variant (23) and the alignment illustrated in Fig. S3. *B*, midlevel optical sections of tsA201 cells transfected with GFP-Ca_V_2.1 Δ2-like variant, β1b, α2δ1 in combination with either mCherry-JPH3 (*left* column) or mCherry-JPH4 (*right* column). Accumulation of the channel at JPH4-induced junction is visible (*white* arrowheads). *C*, Pearson's colocalization coefficients for the specified combinations of Ca_V_2.1 and junctophilins, calculated from optical sections of the *bottom* of the cell. Pearson’s coefficients for Ca_V_2.1 with the intact synprint domain are part of a data set previously described in Perni and Beam, 2021 (21). All data are reported as values from individual cells (*circles*), with the mean indicated by the height of the superimposed rectangles, ± SD [2-way ANOVA: “no JPH v.s. JPH3”: *p* = 0.93, F (1,110) = 0.008. 2-way ANOVA “no JPH” v.s. “JPH4”, *p* < 0.0001, F (1, 111) = 172.9; *post hoc* Sidak's test: ∗∗*p* = 0.0035, n.s. *p* = 0.135]. *D*, percentage of peak Ca^2+^ current remaining 700 ms after the peak (I_700_/I_peak_) is plotted (mean ± SD) as a function of test potential in tsA201 cells transfected with GFP-tagged Ca_V_2.1 Δ2-like variant, β1b and α2δ1 together with either no junctophilins (*black*), JPH3 (*blue*), or JPH4 (*gold*), which were tagged with mCherry. The inset illustrates representative Ca^2+^ currents (scaled to match in amplitude) elicited by an 800 ms depolarization to +50 mV, with the *vertical* dotted line indicating the current 700 ms after the peak. On average, the inactivation of the Ca_V_2.1 Δ2-like variant was unaffected by JPH3 but significantly reduced by JPH4 (2-way ANOVA, *post hoc* Sidak's test: ∗*p* = 0.032; ∗∗*p* = 0.002; ∗∗∗∗*p* < 0.0001). Numbers in parentheses indicate total number of analyzed cells/number of separate transfected dishes. Bars represent 2 μm.

The observation that JPH3_1:707_ colocalizes only with the proximal, synprint-containing segment of the II-III loop, whereas JPH4_1:576_ colocalizes with both the proximal and distal segments of the loop, raises the question of whether this difference might be important for interactions between the full-length JPHs and Ca_V_2.1 isoforms with an altered synprint region. In this regard, it is noteworthy that neuroendocrine cells and most brain regions in rats have been found to contain mRNAs encoding two Ca_V_2.1 splice variants with a deletion of either 194 (Ca_V_2.1–Δ1) or 155 (Ca_V_2.1-Δ2) amino acids in the center of the synprint domain (23). Because current amplitudes were reduced ∼95% for the Δ1 isoform but nearly normal for Δ2 isoform (23), we created a complementary DNA construct that encoded rabbit GFP-Ca_V_2.1 lacking the region of the II-III loop corresponding to that deleted in rat Ca_V_2.1-Δ2 (Figs. 4*A*, S3).

Confocal images of this construct, "Ca_V_2.1 Δ2-like," expressed in tsA201 cells together with β1b, α2-δ1, and either mCherry-JPH3 or mCherry-JPH4 (Fig. 4*B*), were used to compute the Pearson’s coefficients which are compared, in Figure 4*C*, with those we previously obtained for Ca_V_2.1 versus JPH3 or JPH4 (21). For JPH3, the mean Pearson’s coefficient for Ca_V_2.1 Δ2-like (0.26) was significantly smaller than that for Ca_V_2.1 with an intact synprint domain (0.49). For JPH4, the mean for Ca_V_2.1 Δ2-like (0.42) was somewhat smaller than for Ca_V_2.1 (0.53), but this difference was not statistically significant (Fig. 4*C*). Because our previous work had shown that both JPH3 and JPH4 caused a >2-fold slowing of the inactivation of currents *via* Ca_V_2.1 channels containing an intact synprint domain (21), we next compared the abilities of JPH3 and JPH4 to slow the inactivation of the Ca_V_2.1 Δ2-like channel. Figure 4*D* plots the extent of inactivation as the ratio of the current amplitude at 700 ms relative to the peak current (I_700_/I_peak_) as a function of voltage. Unlike its effect on Ca_V_2.1 with an intact synprint domain, JPH3 had no effect on the inactivation of the Ca_V_2.1 Δ2-like channel (compare blue and black data points in Fig. 4*D*). By contrast, JPH4 caused a large slowing of the inactivation of the Ca_V_2.1 Δ2-like channel (gold data points, Fig. 4*D*). Thus, the synprint region of the II-III loop appears to be important for the ability of JPH3 to recruit Ca_V_2.1 to ER–PM junctions, and essential for its ability to slow inactivation. However, because JPH4 can interact with the distal segment of the II-III loop, it is able to both recruit to junctions and slow the inactivation of a Ca_V_2.1 variant that has a large deletion within the synprint domain.

### C-terminal truncation that removes all or most of the **α**-helical domain abolishes colocalization of JPH3 and JPH4 to the Ca_V_2.1 II-III loop

**Figure 5:**
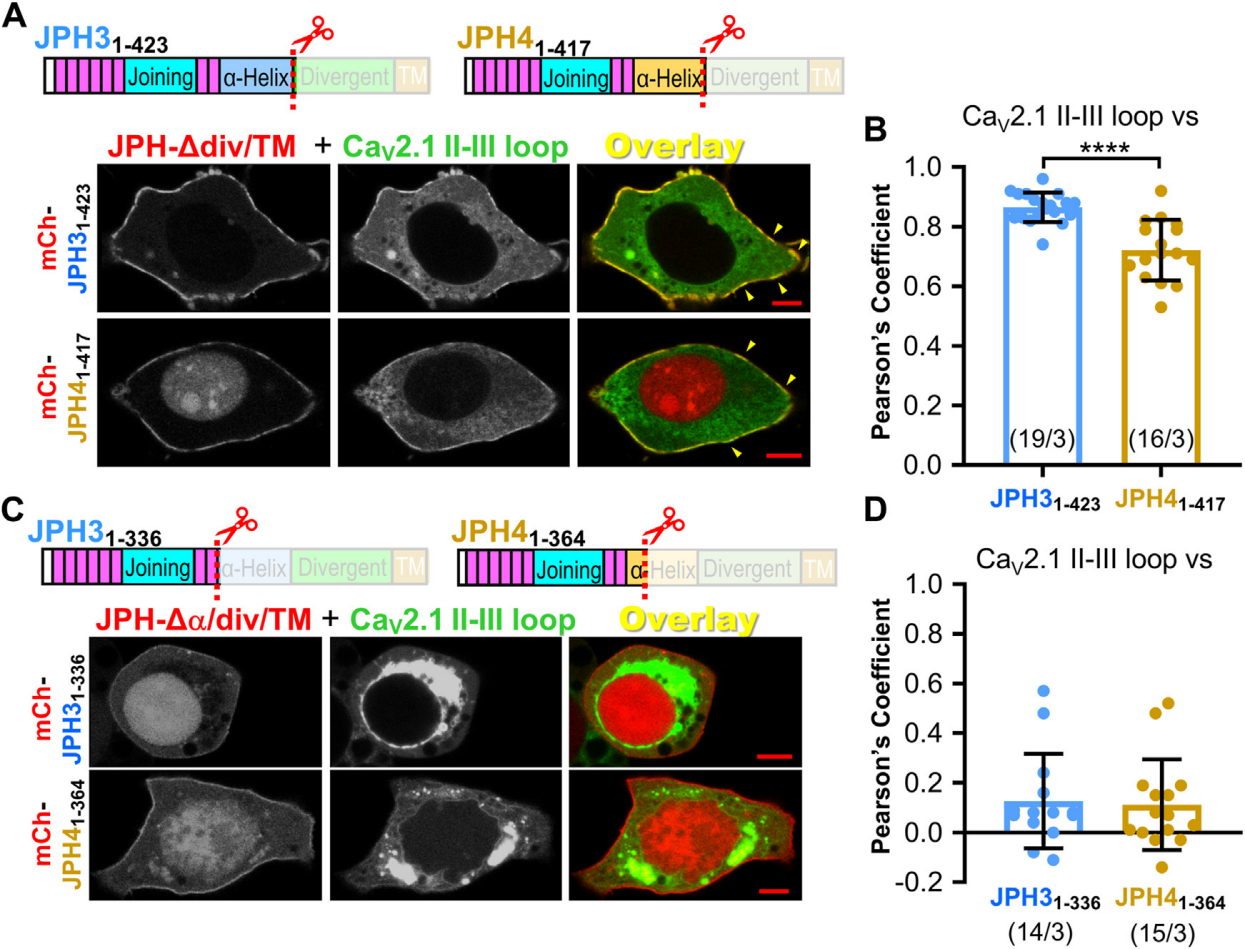
**JPH3 and JPH4 constructs lacking the divergent and TM domains interact with the Ca_V_2.1 II-III loop, but this interaction is lost after the additional removal of all or most of the α-helical domain.***A*, schematic representation of C-terminally truncated JPH constructs JPH3_1-423_ and JPH4_1-417_, in which the divergent and transmembrane domains have been removed and midlevel optical sections of tsA201 cells expressing either of these two mCherry-tagged constructs in combination with GFP-tagged Ca_V_2.1 II-III loop. The II-III loop colocalized with both JPH3_1-707_ and JPH4_1-576,_ as indicated by the *yellow* segments at the cell periphery in the overlaid images (*e.g.*, *yellow* arrowheads). Pearson's coefficients for these construct combinations, calculated from *bottom*-surface scans, are plotted in (*B*). *C*, schematic representation of JPH3_1-336_ and JPH4_1-364_ in which C-terminal truncation removed all, or part, respectively, of the α-helical domain and midlevel optical sections of tsA201 cells expressing mCherry-JPH3_1-336_ or mCherry-JPH4_1-364_ together with the GFP-tagged Ca_V_2.1 II-III loop. There was no apparent colocalization of the II-III loop with either of these constructs. Pearson's coefficients, calculated from bottom-surface scans, are plotted in (*D*). In (*B*) and (*D*), *circles* indicate values for individual cells, with the mean ± SD indicated by the superimposed rectangle and horizontal *black* lines, respectively. Numbers in parentheses indicate total number of analyzed cells/number of separate transfected dishes. ∗∗∗∗*p* < 0.0001; n.s. *p* = 0.84 (*t* test with Welch's correction). Bars represent 5 μm in (*A*) and (*C*).

Having found that JPH3 and JPH4 recruit the Ca_V_2.1 II-III loop to ER–PM junctions, we next attempted to identify domains of these JPHs involved in this recruitment. Depending on the binding partner, previous work had implicated disparate regions of the JPHs, ranging from an interaction with RyRs (21) involving the JPH divergent domain, to an interaction with Ca_V_1.1 (18) which involved the JPH MORN and α-helical domains. Thus, as a simple approach for identifying regions important for colocalization with the Ca_V_2.1 II-III loop, we tested JPH constructs with C-terminal deletions of increasing size. The constructs JPH3_1-423_ and JPH4_1-417_, in which both the divergent and TM domains were deleted, retained the ability to cause the accumulation of the Ca_V_2.1 II-III loop at the cell surface with a distribution overlapping that of the JPH constructs (Fig. 5*A*). The Pearson's coefficients (Fig. 5*B*) indicated that the colocalization was slightly greater for JPH3_1-423_ than for JPH4_1-417_. A possible explanation for this difference is that a substantial fraction of JPH4_1-417_ trafficked to the cell nucleus (*cf.* Fig. 5*A*), leaving a lesser amount of protein available at the cell surface to interact with the pool of nearby Ca_V_2.1 II-III loop.

Because the Ca_V_2.1 II-III loop still colocalized with JPH constructs lacking the divergent and TM domains, we next tested whether this would still occur for JPH constructs with a larger C-terminal truncation that additionally deleted all (JPH3_1-336_) or most (JPH4_1-364_) of the α-helical domain. After this additional deletion, the constructs tended to accumulate in the nucleus so that a clear association of JPH3_1-336_ or JPH4_1-364_ with the PM was only found in a subset of transfected cells (Fig. 5*B*). In such cells, the Ca_V_2.1 II-III loop did not colocalize with either JPH3_1-336_ or JPH4_1-364_ (Fig. 5, *C* and *D*). This result is consistent with the hypothesis that the α-helical domain is required for recruitment of the II-III loop, although the possibility cannot be excluded that the loss of colocalization is a consequence of the deletion causing altered structure in the upstream MORN and joining domains.

### The differential association of JPH3 and JPH4 with the distal segment of Ca_V_2.1 II-III loop depends on the **α**-helical domain

**Figure 6:**
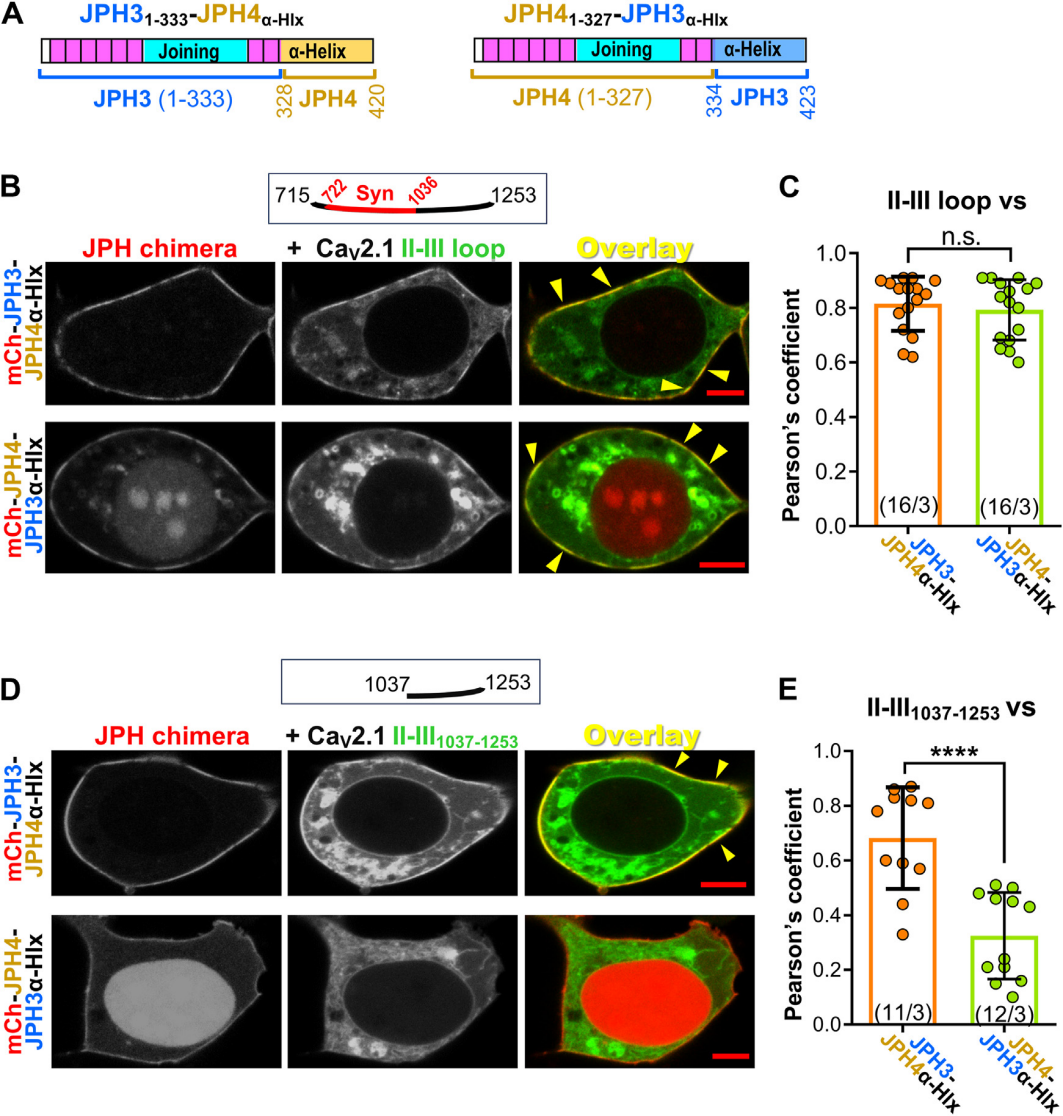
**The α-helical domain is responsible for the ability of JPH4 to interact with the distal half of the Ca_V_2.1 II-III loop.***A*, schematic representation of constructs JPH3_1-333_-JPH4_α-Hlx_, which contains the MORN and joining domains of JPH3 fused with the α-helical domain of JPH4, and the complementary construct JPH4_1-327_-JPH3_α-Hlx_, containing the MORN and joining domains of JPH4 and the α-helical domain of JPH3. The first and last residues of the swapped α-helical domains are indicated in *gold* (JPH4 α-helical domain) and *blue* (JPH3 α-helical domain). *B*, midlevel optical sections of tsA201 cells expressing mCherry-tagged JPH3_1-333_-JPH4_α-Hlx_ or JPH4_1-327_-JPH3_α-Hlx_ together with the full-length Ca_V_2.1 II-III loop tagged with GFP. The full-length II-III loop colocalized at the surface with both these junctophilin chimeras (*yellow* segments in overlays, some indicated by arrowheads). Pearson's coefficients for these construct combinations, calculated from bottom-surface scans, are plotted in (*C*). *D*, midlevel optical sections of tsA201 cells expressing mCherry-tagged JPH3_1-333_-JPH4_α-Hlx_ or JPH4_1-327_-JPH3_α-Hlx_ together with the distal half of the Ca_V_2.1 II-III loop, II-III_1037-1253_, tagged with GFP. Unlike the full-length II-III loop, which showed clear colocalization with both junctophilin chimeras, the only apparent colocalization of the distal half of the II-III loop was with JPH3_1-333_-JPH4_α-Hlx_. Pearson's coefficients for these combinations of constructs, calculated from bottom-surface scans, are plotted in (*E*). In (*C*) and (*E*), circles indicate values calculated for individual cells, with the mean ± SD indicated by the superimposed rectangle and horizontal *black* lines, respectively. Numbers in parentheses indicate total number of analyzed cells/number of separate transfected dishes. In (*C*) and (*E*), n.s *p* = 0.548; ∗∗∗∗*p* < 0.0001 (*t* test with Welch's correction). Bars represent 5 μm in (*B*) and (*D*).

As another approach to investigating the role of the α-helical domains, we created the chimeric constructs JPH3_1-333_-JPH4_α-Hlx_ (MORN and joining domains of JPH3 followed by the JPH4 α-helical domain) and JPH4_1-327_-JPH3_α-Hlx_ (MORN and joining domains of JPH4 followed by the JPH3 α-helical domain), as illustrated in Figure 6*A*. After expression in tsA201 cells, the full-length Ca_V_2.1 II-III loop colocalized to a similar extent with both JPH3_1-333_-JPH4_α-Hlx_ and JPH4_1-327_-JPH3_α-Hlx_ (Fig. 6*B*) with Pearson's coefficients (Fig. 6*C*) that were comparable to those of JPH3_1-423_ and JPH4_1-417_ (Fig. 5*B*). However, the distal loop, which lacks the synprint domain, colocalized to a much greater degree with JPH3_1-333_-JPH4_α-Hlx_ than with JPH4_1-327_-JPH3_α-Hlx_ (Fig. 6, *D* and *E*). Thus, it appears that the α-helical domain of JPH4, but not that of JPH3, binds to the distal segment of the Ca_V_2.1 II-III loop and is therefore responsible for JPH4’s broader interaction with Ca_V_2.1 II-III loop.

### The **α**-helical domain is not directly responsible for the greater slowing of Ca_V_2.1 inactivation by JPH4 than by JPH3

**Figure 7:**
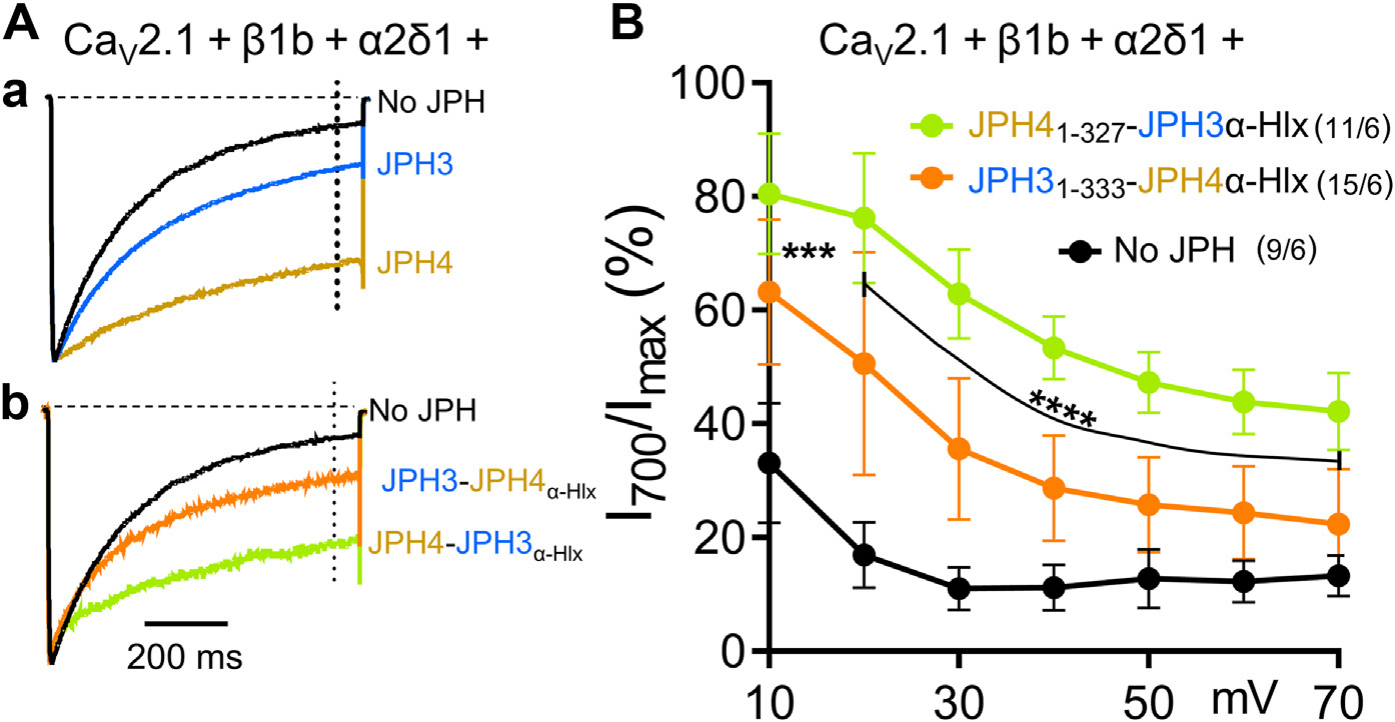
**The greater slowing of Ca_V_2.1 inactivation by JPH4 than by JPH3 is not attributable to the α-helical domain.***A*, representative Ca^2+^ currents (scaled to match in amplitude) elicited by an 800 ms depolarization to +40 mV in tsA201 cells transfected with YFP-Ca_V_2.1, β1b, and α2δ1 together with either no junctophilins (*black*) or with mCherry-tagged JPH3 (*blue*), JPH4 (*gold*), JPH3_1-333_-JPH4_α-Hlx_ (*orange*), or JPH4_1-327_-JPH3_α-Hlx_ (*green*) designated in the figure as JPH3-JPH4_α-Hlx_ and JPH4-JPH3_α-Hlx_, respectively. The vertical *dotted* line indicates the current 700 ms after the peak. *B*, percentage of peak current remaining 700 ms after the peak (I_700_/I_peak_) is plotted (mean ± SD) as a function of test potential for Ca^2+^ currents recorded from tsA201 cells transfected with Ca_V_2.1, β1b, and α2δ1 together with either no junctophilins (*black*), JPH3_1-333_-JPH4_α-Hlx_ (*orange*), or JPH4_1-327_-JPH3_α-Hlx_ (*green*). In (*B*), numbers in parentheses indicate total number of analyzed cells/number of separate transfected dishes. The currents for JPH3 and JPH4, and the current and I_700_/I_peak_ values for no junctophilins, are part of a data set previously described in Perni and Beam, 2021 (21) [2-way ANOVA: “JPH4_1-327_-JPH3α-Hlx v.s. JPH3_1-333_-JPH4α-Hlx”: *p* < 0.0001, F (1,168) = 197.9; *post hoc* Sidak's test: ∗∗∗*p* = 0.0004; ∗∗∗∗*p* < 0.0001].

Taken together, the results described above indicate that JPH3 and JPH4 bind to the Ca_V_2.1 II-III loop and that this binding depends on the JPH α-helical domain. Additionally, our earlier work (21) demonstrated that JPH4 causes a larger decrease in the rate of Ca_V_2.1 inactivation than does JPH3. Thus, the question arises of whether the α-helical domain is responsible for this differential effect on inactivation, which we tested by transfecting tsA201 cells with Ca_V_2.1, the β1b and α2δ1 auxiliary subunits, and either JPH3_1-333_-JPH4_α-Hlx_ or JPH4_1-327_-JPH3_α-Hlx._ As for full-length JPH3 and JPH4 (Fig. 7*Aa*), inactivation of Ca^2+^ currents *via* Ca_V_2.1 was slowed by both JPH3_1-333_-JPH4_α-Hlx_ and JPH4_1-327_-JPH3_α-Hlx_ (Fig. 7*Ab*). The slowing of inactivation was greater for JPH4_1-327_-JPH3_α-Hlx_ (Fig. 7*B*) even though this construct contained the α-helical domain of JPH3. Thus, it appears that the greater slowing of inactivation by full-length JPH4 cannot be attributed to its α-helical domain and that the upstream MORN and/or joining domains play a role in regulating the inactivation of Ca_V_2.1.

## Discussion

Here, we have used electrophysiology and expression of fluorescently tagged constructs in tsA201 cells to identify the protein domains that are important for the ability of the neuronal JPHs, JPH3 and JPH4, to recruit Ca_V_2.1 to ER–PM junctions and to slow the channel’s inactivation. As one approach, we expressed each of the five Ca_V_2.1 cytoplasmic domains (N-terminus, I-II loop, II-III loop, III-IV loop, C-terminus) N-terminally tagged with EGFP, together with JPH3 or JPH4, N-terminally tagged with mCherry. The Ca_V_2.1 II-III loop showed substantial colocalization with both JPH3 and JPH4, whereas none of the other cytoplasmic domains colocalized with either JPH3 or JPH4 (Fig. 1). Concordant results were obtained when we co-expressed JPH3 with constructs in which the Ca_V_2.1 cytoplasmic domains were linked to the carboxy terminal of GFP-(Ca_V_1.2 I-II loop): colocalization with JPH3 only occurred for the construct containing the Ca_V_2.1 II-III loop (Fig. S1, *A*–*G*). Thus, the II-III loop may be the primary site of Ca_V_2.1 that governs its recruitment by the neuronal JPHs. This contrasts with the interaction between the JPHs (JPH1 and JPH2) and L-type Ca^2+^ channels (Ca_V_1.1 and Ca_V_1.2) that are expressed in striated muscle, for which the binding of JPH1 and JPH2 was found to depend on a region within the proximal segment of the channels’ C-terminus (17, 18).

To determine whether the colocalization of the Ca_V_2.1 II-III loop with JPH3 and JPH4 depended on the ability of these JPHs to form ER–PM junctions, we co-expressed the GFP-tagged loop with JPH constructs (JPH3_1-707_ and JPH4_1-576_), which lack the TM domain that tethers full-length JPH3 and JPH4 to the ER. When expressed alone, JPH3_1-707_ and JPH4_1-576_ had a relatively uniform distribution at the cell surface (Fig. 2*Ba*), unlike the punctate distribution of the ER–PM junctions formed by full-length JPH3 and JPH4 (Fig. 2*Bb*). However, when mCh-JPH3_1-707_ or mCh-JPH4_1-576_ were co-expressed with GFP-(Ca_V_2.1 II-III loop), the loop and truncated JPHs associated with one another to produce an overlapping pattern of green and red puncta (Fig. 2*Bc*). This pattern is consistent with the hypothesis that the association of GFP-(Ca_V_2.1 II-III loop) with both the ER (Fig. S1*I*), and the surface-associated mCh-JPH3_1-707_ or mCh-JPH4_1-576_, was able to induce ER–PM junctions. Judging by its punctate distribution (Fig. S1*H*), the construct GFP-(Ca_V_1.2 I-II loop)-Ca_V_2.1 II-III loop was also able to induce ER–PM junctions because it was associated with both the surface, *via* the I-II loop of Ca_V_1.2 (22), and the ER *via* the (Ca_V_2.1 II-III loop). The mechanism responsible for interaction between the II-III loop and ER membrane is unclear. A search for ER retention signals using SignalP v 5.0 (24) and potential posttranslational modifications using NMT (25), prePS (26), bigPI predictor (27), and MusiteDeep (28) failed to return any obvious sequences or modification sites that would predict such behavior. An alternative possibility is that the Ca_V_2.1 II-III loop binds to an ER-resident protein. This association might involve the C-terminal segment of the II-III loop which appeared to be more strongly associated with the exterior of the ER (Fig. 3*D*) than the synprint-containing, N-terminal half of the loop (Fig. 3*A*).

After finding that the Ca_V_2.1 II-III loop colocalizes with full-length and C-terminally truncated constructs of JPH3 and JPH4, we next tested subdivisions of the loop. We found that the N-terminal half of the loop (residues 715–1084) colocalized with the C-terminally truncated constructs of both JPH3 and JPH4 (Fig. 3, *A*–*C*). By contrast, the C-terminal half of the II-III loop (residues 1037–1253) colocalized only with the JPH4 construct (Fig. 3, *D*–*F*). Significantly, the N-terminal half of the II-III loop contains the synprint site (4), which is responsible for binding to syntaxin and synaptotagmin 1; these interactions appear to be important for Ca_V_2.1 localization to presynaptic nerve terminals and for neurotransmitter release from these terminals (29). Much like syntaxin, for which strong binding did not occur with partial segments of the synprint domain (4), colocalization with JPH3 and JPH4 constructs was lost for partial segments of the synprint domain (Fig. S2).

The colocalization of the synprint-containing, N-terminal half of the Ca_V_2.1 II-III loop with both JPH3_1-707_ and JPH4_1-576_, and of the C-terminal half of the loop only with JPH4_1-576_ (Fig. 3), raised the possibility that a Ca_V_2.1 channel with an altered synprint region might behave differentially with respect to full-length JPH3 and JPH4 and that this might be functionally important. To examine this possibility, we constructed a rabbit Ca_V_2.1 with a deletion in the center of the synprint region that corresponded to a splice variant (Δ2) of rat Ca_V_2.1 (Fig. S3). For full-length JPH3, but not JPH4, colocalization was significantly reduced for Δ2-like rabbit construct compared to Ca_V_2.1 with an intact synprint region (Fig. 4, *B* and *C*). Unlike Ca_V_2.1 with an intact synprint domain, for which inactivation was slowed by both JPH3 and JPH4 (21), inactivation of the Δ2-like variant of Ca_V_2.1 was only slowed by JPH4 (Fig. 4*D*). The effects on inactivation, taken together with the results on colocalization between Ca_V_2.1 cytoplasmic domains and the JPHs (Figure 1, Figure 2, Figure 3, S1 and S2), support the conclusion that JPH3 and JPH4 both interact with nonspliced Ca_V_2.1 and that this interaction occurs *via* the synprint domain for JPH3 or *via* either the synprint domain or C-terminal half of the loop for JPH4. Generalizing, one would expect that naturally occurring splice variants of Ca_V_2.1, in which portions of the synprint domain are deleted, such as rat brain Δ1 and Δ2 (23), would be recruited to ER–PM junctions containing JPH4 but not to those containing only JPH3. Additionally, neurological disorders linked to mutations within the synprint region of Ca_V_2.1 might partly arise from altered interactions with JPH3.

The JPH α-helical domain appeared to play a key role in the colocalization of the isolated II-III loop of Ca_V_2.1 with the JPHs. In particular, the colocalization of JPH3 and JPH4 constructs with the full-length II-III loop was preserved after deletion of the divergent domain but lost after the additional deletion of all/most of the α-helical domain (Fig. 5). Additionally, a chimeric construct with JPH3 residues 1 to 333 fused to the JPH4 α-helical domain (JPH3_1-333_-JPH4_α-Hlx_) colocalized with the C-terminal half of the II-III loop, whereas the mirror construct, JPH4 residues 1 to 327 fused to the JPH3 α-helical domain (JPH4_1-327_-JPH3_α-Hlx_) did not (Fig. 6). We used these same two chimeric constructs to determine whether the greater slowing of Ca_V_2.1 inactivation by JPH4 than by JPH3 could be attributed to the α-helical domain. However, JPH4_1-327_-JPH3_α-Hlx_ slowed inactivation to a greater extent than JPH3_1-333_-JPH4_α-Hlx_ (Fig. 7), which suggests that regions N-terminal to the α-helical domain are responsible for the greater effect of JPH4 on inactivation.

The interaction of the snap receptor proteins syntaxin and SNAP-25 with the synprint domain of Ca_V_2.1 channels reduces the availability of these channels by causing a hyperpolarizing shift of inactivation (30, 31, 32). By contrast, our previous work (19) and the work we have described here suggests that the binding of JPH3 or JPH4 to the synprint domain increases the availability of these channels by slowing the rate of inactivation. The opposing effects on the inactivation of Ca_V_2.1 provide evidence that interactions solely with the synprint domain cannot account for these effects. Further evidence for this is that JPH4 slows inactivation of a Ca_V_2.1 construct lacking a large segment of the synprint domain (Fig. 4). Thus, it is reasonable to speculate that both the synprint domain and distal II-III loop of Ca_V_2.1 could serve as docking sites for diverse regulatory proteins, which can then modulate function by interacting with other regions of the channel.

In studies using transfected cells, it is important to consider the possible role of endogenously expressed proteins. In this regard, it is of obvious relevance that earlier work demonstrated that the expression of human α_1A_ (P/Q type) channels in HEK293 cells upregulated the production of syntaxin-1A resulting in a ∼10 mV hyperpolarizing shift of inactivation (33). For several reasons, we think that syntaxin1A was not a confounding factor in our experiments. First, the work of Sutton *et al.* demonstrated that the expression of syntaxin-1A depended on the entry of extracellular Ca^2+^ *via* the expressed P/Q channels, due to a low level of tonic activation at the resting potential of about −30 mV in HEK293 cells. It seems unlikely that such tonic activation occurred for the Ca_V_2.1 (P/Q type) channels used in our experiments because these channels (BI-2) have a peak I-V relationship (34) that is shifted ∼25 mV in the depolarized direction compared to that of the α_1A_ channels used by Sutton *et al.* [inset to their Fig. 1*A*, (33)]. We cannot pinpoint the reasons for this difference in behavior, but it is known that alternative splicing significantly affects the behavior of Ca_V_2.1 [(*e.g.*, Bourinet *et al.*, 1999 (35)]. Second, in our experiments, similar current amplitudes were found when Ca_V_2.1 was expressed with or without JPH3 or JPH4 (21), which is evidence against the idea that these JPHs are antagonizing an inhibitory effect of syntaxin-1A. Third, the observation that JPH4 slows the inactivation of the Ca_V_2.1 Δ2-like construct that lacks a large segment of the synprint domain (Fig. 4*D*) cannot be easily explained by a competition with syntaxin-1A for binding to the synprint domain.

In neurons, direct competition between syntaxin-1A and the JPHs for binding to the synprint site of Ca_V_2.1 would only occur if these proteins were localized in the same subcellular compartments. This seems unlikely in presynaptic terminals, where ER–PM junctions containing JPHs are not known to occur. Conversely, Ca_V_2.1 and the neuronal JPHs appear to be primarily present in proximal and medial dendrites of hippocampal neurons (11) and in cell body and proximal dendrites of cerebellar Purkinje cells (6), whereas syntaxin-1A is concentrated in presynaptic terminals (36).

The ER–PM junctions induced by the JPHs can contain diverse channels in both the PM and ER (37, 38) and the many factors that control which ones are present are only partially understood. For example, the initial MORN domains of JPH2 (those N-terminal to the joining domain) were found to interact with small-conductance calcium-activated potassium channels (39) and with a C-terminal segment of Ca_V_1.1 (18). However, the joining domain of JPH2 was also shown to be important for the interaction with Ca_V_1.2 (40), and the joining domain of JPH1 was present in a peptide that interacted with Ca_V_1.1 (16). The work reported here indicates that the interaction with Ca_V_2.1 involves regions of JPH3 and JPH4 located C-terminally to those discussed above, namely the α-helical domains. Further towards the C-terminal, the divergent domain was found to be important for the ability of JPH3 to recruit all three RyR isoforms to ER–PM junctions (21), but not for the junctional recruitment of Ca_V_2.1 (Fig. 5*A*). Thus, interactions of PM and ER channels with different segments of the JPHs may help to stabilize the presence of multiple channel types in ER–PM junctions.

**Figure 8:**
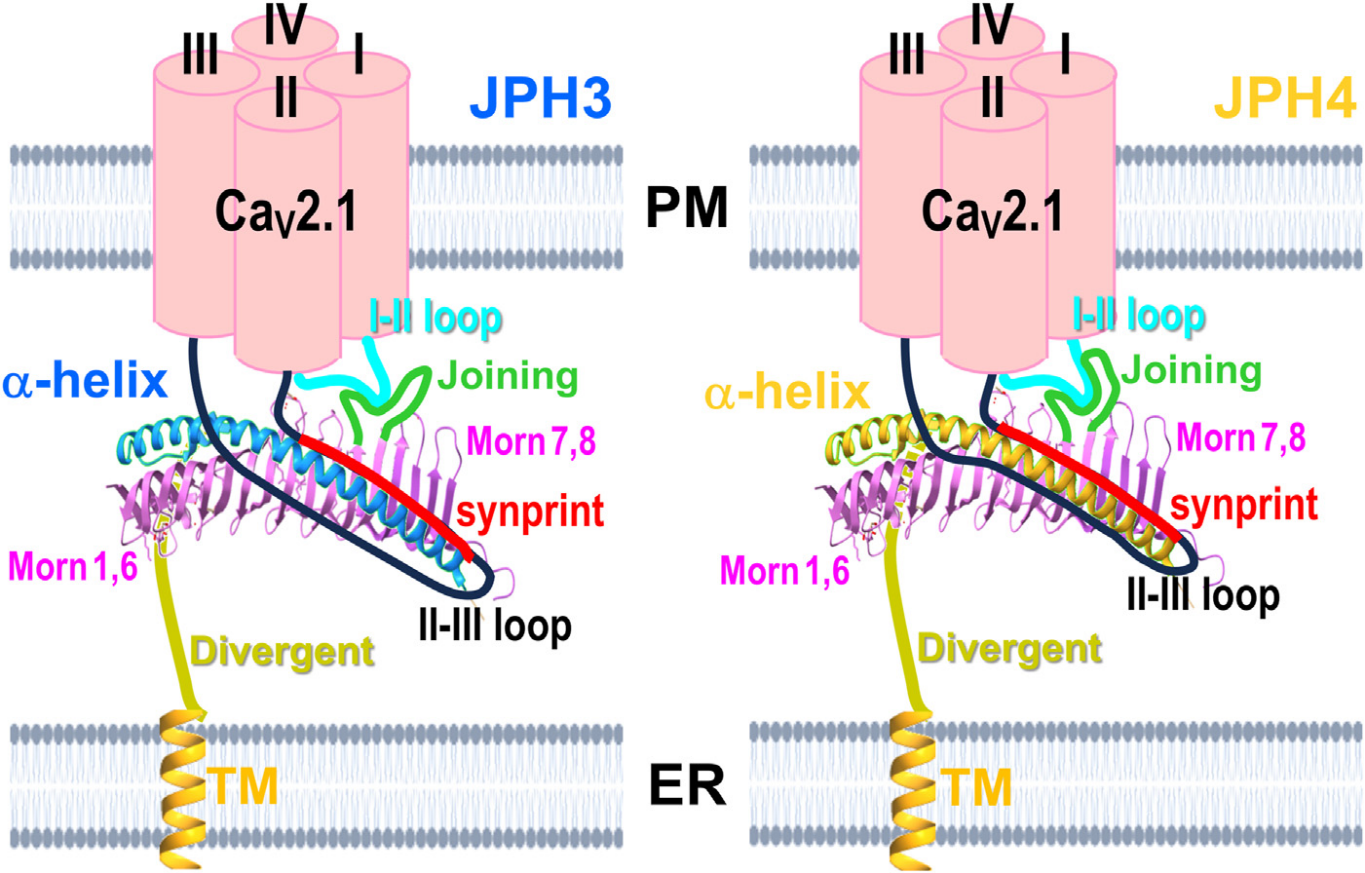
**Summary model of interactions between Ca_V_2.1 and the neuronal junctophilins.** The MORN and α-helical domains are represented according to the crystal structure that was determined for a JPH1 construct encompassing these domains linked by a greatly abbreviated joining domain (PDB: 7rw4). The structure of the joining, divergent, and transmembrane domains has not yet been experimentally determined. In the model, the α-helical domains of both JPH3 (*left*, in *blue*) and JPH4 (*right*, in *yellow*) bind to the proximal segment of the Ca_V_2.1 II-III loop (in *black*), which contains the synprint domain (in *red*). The α-helical domain of JPH4 also interacts with the distal half of the II-III loop. These interactions with the II-III loop cause Ca_V_2.1 to localize at ER-PM junctions induced by both JPH3 and JPH4. Because JPH3 requires the synprint domain for its interaction with Ca_V_2.1, Ca_V_2.1 variants having significant deletions in the synprint domain will interact with JPH4 but not with JPH3. Because regions N-terminal to the α-helical domain appear to be involved in the greater slowing of Ca_V_2.1 inactivation by JPH4 than by JPH3 (Fig. 7), the model indicates a possible interaction of the joining domain (in *green*) with the I-II loop of Ca_V_2.1 (in *cyan*). Note that for clarity of presentation, the crystallographic structure is shown separated from the plasma membrane and oriented with the α-helical domain facing the viewer. The model is equally consistent with an orientation in which the structure is rotated around its long axis such that one or more MORN domains could interact with the plasma membrane.

Figure 8 presents a schematic model that summarizes the interactions we have found between Ca_V_2.1 and the neuronal JPHs. As a starting point, the model uses the crystal structure determined for a JPH1 construct (18) consisting of MORN domains 1 to 6, a shortened joining domain, MORN domains 7 to 8, and the α-helical domain. The regions that have no determined structure are indicated in green (joining domain), olive (divergent domain), and orange (TM domain). In the model, the α-helical domain of both JPH3 and JPH4 binds to the synprint domain of the Ca_V_2.1 II-III loop. The α-helical domain of JPH4, but not that of JPH3, additionally binds to the C-terminal half of the II-III loop. To account for the greater slowing of inactivation by the chimera JPH4_1-327_-JPH3_α-Hlx_ than by the chimera JPH3_1-333_-JPH4_α-Hlx_, the model postulates an additional contact between the joining domain and the I-II loop of Ca_V_2.1: this contact region is more extended for JPH4 as an indication of its larger effect on inactivation. However, it is equally possible that, on the JPH side, one or more MORN domains are involved and that, on the Ca_V_2.1 side, regions other than the II-III loop are involved.

### Conclusions

In this work, we investigated the regions involved in the interaction between the neuronal JPHs JPH3 and JPH4 and the P/Q-type voltage-gated calcium channel Ca_V_2.1. We found that the II-III loop of Ca_V_2.1 is essential for the recruitment of the channel into JPH-induced ER–PM junctions and the modulation of Ca_V_2.1 inactivation by these JPHs. Specifically, the Ca_V_2.1 II-III loop contains two binding sites for JPH. The first site, located in the N-terminal half of the loop, can bind to both JPH3 and JPH4 and includes the synprint domain responsible for interactions of the channel with presynaptic proteins. The second site is in the C-terminal half of the loop and exclusively interacts with JPH4. On the JPH side, the α-helical domain is essential for interactions with the II-III loop and dictates the isoform-specific binding of JPH4 to the C-terminal half of the loop, but it is not responsible for the stronger effect of JPH4 on Ca_V_2.1 inactivation, suggesting the existence of another interaction between JPHs and Ca_V_2.1 that regulates this functional effect.

## Experimental procedures

### Expression plasmids

JPH3, JPH4: The complementary DNAs encoding mCherry-JPH3, mCherry-JPH4, mCherry-JPH3_1-707_, and mCherry-JPH4_1-576_ were described earlier (21). mCherry-JPH3_1-423_ was created using the primers,

Fw: CGAGCTCAAGCTTCGAATTCTGC

Rv: TCCCGGGATCCGAAGGAAGGGG

They are designed to amplify the JPH3 coding sequence of mCherry-JPH3, starting from the BamHI site 5′ to the JPH3 start codon, and extending through the codon-encoding residue F423 with the addition of a second BamHI site at the 3′ of the amplified fragment. This fragment was then digested with BamHI and ligated at the 3′ end of the mCherry coding sequence in the plasmid mCherry-C1, described earlier (21), cut with the same enzyme.

mCherry-JPH4_1-416_ was created using the primers,

Fw: CGAGCTGAATTCCGCCACCATGTCC

Rv: CTAGCATGGGTACCAGGTCCTGG

They are designed to amplify a fragment of mCherry-JPH4 encoding resides 1 to 416 of JPH4 and to add an EcoRI and a KpnI restriction site at the 5′ and 3′ ends of the amplified fragment, respectively. mCherry-C1 and the amplified fragment were then digested with these two enzymes and ligated to obtain the final construct.

mCherry-JPH3_1-336_ was created using the primers,

Fw: CGAGCTCAAGCTTCGAATTCTGC

Rv: TCTGCTTGCAATTGCCCTCCTCCTTGG

designed to amplify the JPH3 coding sequence starting from the KpnI site 5′ to the start codon and extending through the codon for residue G336 with the addition of a MfeI site at the 3′ end of the amplified fragment. The fragment was then digested with KpnI and MfeI and ligated at the 3′ end of the mCherry coding sequence in mCherry-C1, cut with the same enzymes.

mCherry-JPH4_1-364_ was created using the primers.

Fw: CGAGCTCAAGCTTCGAATTCTGC

Rv: CGGGCGCAATTGACAGCCCTGTCC

They are designed to amplify the JPH4 coding sequence starting from the KpnI site at the 5′ to the start codon and extending through the codon for residue V364, with the addition of an MfeI site at the 3′ end of the amplified fragment. The fragment was then digested with KpnI and MfeI and ligated at the 3′ end of the mCherry coding sequence in mCherry-C1, cut with the same enzymes.

mCherry-JPH3_1-333_-JPH4_α-Hlx_ and mCherry-JPH4_1-327_-JPH3_α-Hlx_ were created using the Gibson assembly method. The primers used for mCherry-JPH3_1-333_-JPH4_α-Hlx_ were

Vector Fw: CCCATGCTATGATAAACCCGCTGATCAGCC

Vector Rv: CCCTCCTCCTTGGTGCCGTC

Fragment FW: CACCAAGGAGGAGGGCAAGTACAAGCG

Fragment Rv: GGGTTTATCATAGCATGGGCTGCAGGT

mCherry-JPH3 was used as template for the vector, and mCherry-JPH4 was used as a template for the fragment. The set of plasmids was designed to substitute the JPH4 α-helical domain (residues 328–420) in place of the C-terminal part of JPH3 that included residues from 334 to the terminal codon.

The primers used for mCherry-JPH4_1-327_-JPH3_α-Hlx_ were

Vector Fw: CCTTCCTTCTGATAAACCCGCTGATCAGCCT

Vector Rv: CCCTCCTCGCGGGAGCCGTC

Fragment FW: CTCCCGCGAGGAGGGCAAGTACAAGCAG

Fragment Rv: GGGTTTATCAGAAGGAAGGGGAGAACTCTTTGG

mCherry-JPH4 was used as template for the vector, and mCherry-JPH3 was used as a template for the fragment. The set of plasmids was designed to substitute the JPH3 α-helical domain (residues 334–423) in place of the C-terminal part of JPH4 that included residues from 328 to the terminal codon.

### Voltage-gated channels and subunits

EYFP-Ca_V_2.1, α2-δ1, and β1b were previously described (21). Each of the EGFP-tagged intracellular domains of Ca_V_2.1 was created by PCR amplification using the EGFP-Ca_V_2.1 construct (21) as a template, and the following primers designed to add a SalI and a BamHI restriction site at the 5′ and 3′ ends, respectively, of each intracellular domain fragment.

N-terminus: Fw: GCAGTCGACATGGCCCGTTTCG

Rv: GGCGGATCCCTAAGGCCATTCGGTGATCTTC

I-II loop: Fw: GCAGTCGACGGGGAGTTTGCCAAAGAAAGG

Rv: GGCGGATCCCTACTGAGTTTTGACCATGCGAC

II-III loop: Fw: GCAGTCGACAACCTGGCCAATGCCCAGGAG

Rv: GGCGGATCCCTAGCGCAGGTTCAGGATGTAATG

III-IV loop: Fw: GCAGTCGACATCACCTTCCAGGAGCAGGGC

Rv: GGCGGATCCCTACGGGGACACCACGAACTGC

C-terminus: Fw: GCAGTCGACAACTTCGAGTACCTCACGCGC

Rv: GGCGGATCCCTAGGGGGAGGGGGCGCTGGCTC

Each amplified fragment was inserted at the 3′ end of the EGFP coding sequence in the pEGFP-C1 plasmid (Clontech) digested with SalI and BamHI.

### II-III loop fragments

GFP-Ca_V_2.1_715-1084_ was created by cutting the sequence starting from GFP and ending at Ca_V_2.1 residue 1084 out of the EGFP-Ca_V_2.1 II-III loop construct using the restriction enzyme NheI. The excised fragment is then pasted into the multiple cloning site of a pcDNA3 plasmid (Invitrogen) digested with XbaI.

GFP-Ca_V_2.1_1037-1253_ was created by amplification of the sequence encoding Ca_V_2.1 residues 1037 to 1253, using the EGFP-Ca_V_2.1 II-III loop plasmid as a template with the primers:

Fw: AGGGAAGCTTGGCACCGGAGGAG

Rv: CCAAACTGGAACAACACTCAACCC

They are designed to add a HindIII restriction site at the 5′ end of residue 715 and a BamHI restriction site 3′ to the stop codon after residue 1253. The fragment was then inserted in place of the entire II-III loop sequence in the EGFP-Ca_V_2.1 II-III loop construct using HindIII and BamHI.

GFP-Ca_V_2.1 II-III_715-909_ was created by cutting the sequence encoding for Ca_V_2.1 residues 715 to 909 from the GFP-Ca_V_2.1_715-1084_ plasmid with EcoRI and XmaI. The fragment was ligated into the pEGFP-C1 plasmid cut with the same enzymes.

GFP-Ca_V_2.1 II-III_909-1084_ was created by excising the sequence encoding Ca_V_2.1 residues 715 to 908 from construct GFP-Ca_V_2.1_715-1084_, using the enzymes BsrGI and BsiWI, and ligating the recircularized resulting vector.

Ca_V_2.1 Δ2-like: To create this construct, in which a segment of the Ca_V_2.1 II-III loop has been deleted, the sequence encoding residues 1 to 1965 of rabbit Ca_V_2.1 (Accession # 1709354B) was excised from the YFP-Ca_V_2.1 plasmid and inserted into a pCEP4 (Invitrogen) empty vector using the restriction enzyme BglII. This vector was then used as a template for the amplification of two fragments corresponding to the entire BglII fragment but without the sequence that is deleted in the Δ2-like splice variant. The fragment at the 5′ end of the deletion was amplified using the following primers:

Fragment 1, Fw: AGAACTGGTAGGTATGGAAAGATCTCGAGCTCAAGC

Fragment 1, Rv: CCCTCGCGGCTGGCCAGCAG

The fragment at the 3′ end of the deletion was amplified using the primers:

Fragment 2, Fw: GGCCAGCCGCGAGGGCAGCCGG

Fragment 2, Rv: GGTACCCAGCTTCTAGAAGATCTTGCCCACT

These two amplified fragments were then ligated in place of the original Ca_V_2.1 BglII fragment in the pCEP4 vector by Gibson assembly. The new Ca_V_2.1 Δ2 BglII fragment was then cut from the pCEP4 vector and inserted into the YFP-Ca_V_2.1 plasmid construct using BglII to create the final YFP-Ca_V_2.1 Δ2-like construct.

### Cell culture and complementary DNA transfection

tsA201 cells (ECACC 96121229) were cultured in high-glucose Dulbecco's Modified Eagle Medium (Mediatech), supplemented with 10% (vol/vol) fetal bovine serum and 2 mM glutamine in a humidified incubator with 5% (vol/vol) CO_2_. Cells at ∼70% confluency were transfected by exposure for 3.5 h to the jetPRIME reagent (Polyplus-transfection Inc.) containing either 1 μg (Ca_V_2.1) or 0.5 μg (Ca_V_2.1 domains and fragments, β1b, α2δ1, and JPH constructs) per 35 mm plastic culture dish (Falcon). After the 3.5 h of transfection, the cells were rinsed, detached from the dish using trypsin-EDTA (Mediatech), and replated at ∼1.5 × 10^4^ cells/dish in 35 mm plastic culture dishes for electrophysiology or at ∼2.5 × 10^4^/cm^2^ in glass-bottomed microwell dishes (MatTek, 35 mm dish, 14 mm microwell diameter), previously coated with collagen type III (Sigma-Aldrich) or ECL (Millipore), for confocal imaging. Electrophysiological data were recorded 48 h after transfection; imaging data were recorded 24 h post-transfection.

### Electrophysiology

All experiments were performed at room temperature (∼25 °C). Pipettes were fabricated from borosilicate glass and had resistances of ∼2.5 MΩ when filled with an internal solution consisting of (in mM) the following: 140 Cs-aspartate, 10 Cs-EGTA, 5 MgCl_2_, and 10 Hepes (pH 7.4, with CsOH). The bath solution contained (mM) 145 tetraethylammonium-Cl, 10 CaCl_2_, and 10 Hepes (pH 7.4 with TEA-OH). To record Ca^2+^ currents, cells were held at a potential of −60 mV (to eliminate occasional contamination from T-type currents) and then depolarized to potentials ranging from 0 to +70 mV. Electronic compensation was used to reduce the effective series resistance to < 8 MΩ (time constant < 500 μs). Linear components of leak and capacitive current were corrected with −P/4 online subtraction protocols. Filtering was set at 1 to 2 kHz and digitization at 20 kHz. Channel inactivation was quantified as the percentage of peak current remaining 700 ms after the peak (I_700_/I_peak_).

### Imaging

Cells were superfused with physiological saline (in mM: 146 NaCl, 5 KCl, 2 CaCl_2_, 1 MgCl_2_, 10 Hepes, pH 7.4, with NaOH) and imaged using a Zeiss 710 confocal microscope. Images were obtained as single optical sections (∼0.9 μm thick) with a 63 × (1.4 NA) oil immersion objective. Fluorescence excitation (Ex) and emission (Em) (nanometers) were as follows: GFP (Ex, 488; Em, 493 − 586), YFP (Ex, 514; Em, 515 − 619), mCherry (Ex, 543; Em, 578 − 696). Cells were chosen for analysis solely by the presence of distinguishable surface expression of JPH, regardless of the fluorescence distribution of the co-expressed proteins. Interaction between JPH and Ca_V_2.1 constructs was inferred from colocalization at the cell surface, which was assessed from ∼0.9 μm-thick optical sections either at the cell's substrate-adhering surface or at a level roughly halfway between the bottom and top surfaces of the cell. The cell's substrate-adhering surface was used if the JPH construct was discontinuously distributed at the surface (full-length JPH3 or JPH4) and/or the Ca_V_2.1 construct was discontinuously distributed in the cell interior (full-length or distal half of the II-III loop). When the JPH construct was uniformly distributed at the surface and the Ca_V_2.1 construct was uniformly distributed in the cell interior, mid-level scans were used, excluding the nucleus and obvious protein aggregates from the analysis. Colocalization was quantified using ImageJ and the "Coloc2" plugin which computed the above-threshold Pearson's coefficient with the following settings: threshold regression type = Bisection, PSF = 10, Costes' randomizations = 10.

### Statistical methods

Student's *t* test with Welch's correction was used for comparison between two sets of data. One-way ANOVA, with Tuckey's *post hoc* test, was performed to compare multiple sets of colocalization data. Two-way ANOVA, with Sidak's *post hoc* test was performed for comparison of electrophysiological data.

## Data availability

All the data described in the manuscript are contained within the manuscript. Raw datasets are available upon request from the corresponding author (stefano.perni@unisi.it).

## Supporting information

This article contains supporting information (4, 18, 23).

## Conflict of interest

The authors declare that they have no conflicts of interest with the contents of this article.
