# Supplementary material for: Interaction between CaV2.1 and Junctophilin3/4 depends on the II-III loop of CaV2.1 and on the α-helical region of Junctophilin3/4
Source: J Biol Chem. 2025 Mar 19;301(4):108424. doi: 10.1016/j.jbc.2025.108424 (PMC12017855; doi:10.1016/j.jbc.2025.108424)
Supplement: Supporting information [file mmc1.docx]

**Supporting information**

**
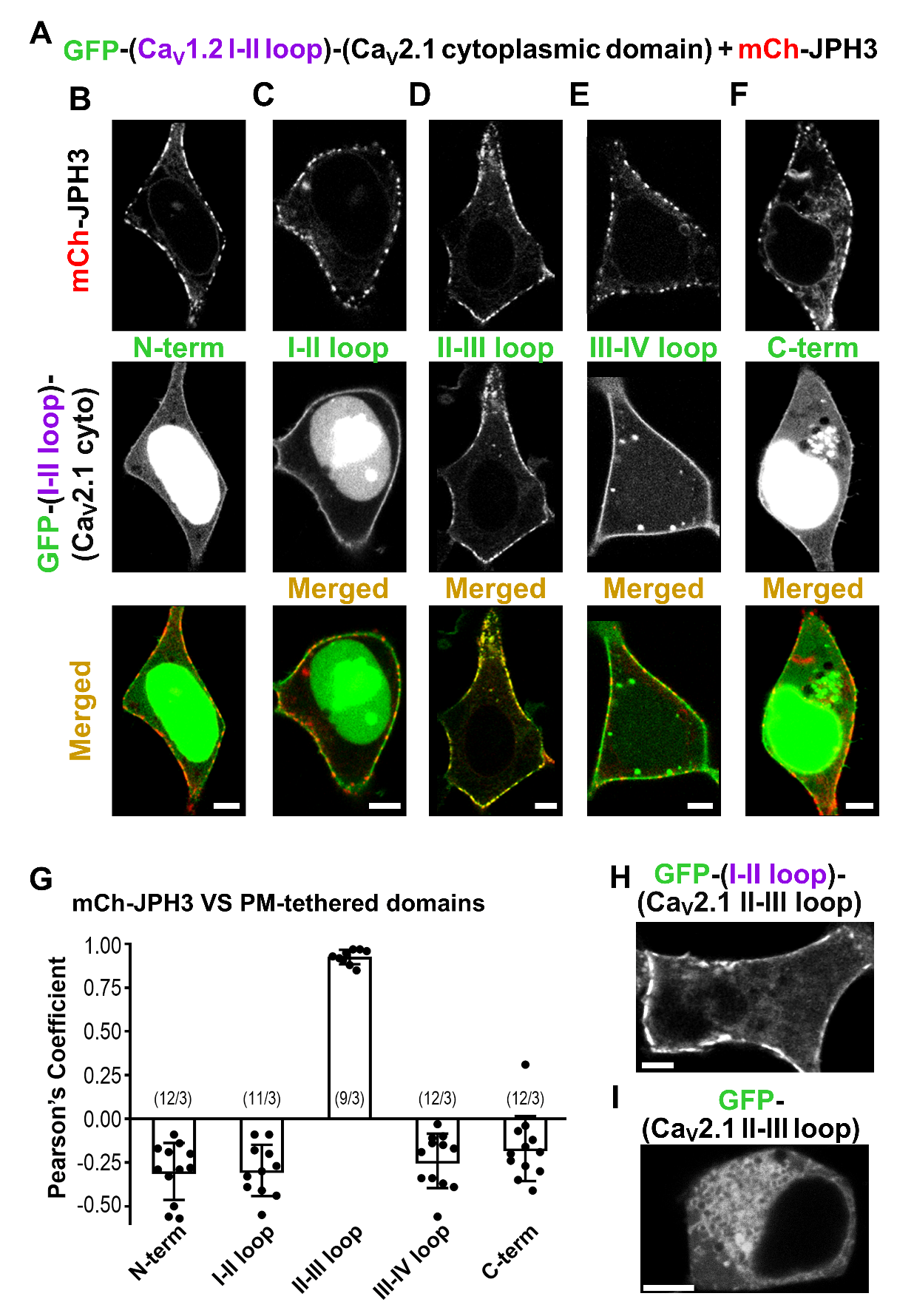
Supplemental Figure 1**

**Supplemental Figure 1. JPH3 interacts with plasma membrane-tethered Ca_V_2.1 II-III loop which can form ER-PM junctions independently of junctophilins.** (A) Schematic representation of the membrane-tethered Ca_V_2.1 cytoplasmic domains. The constructs included a GFP fused to the Ca_V_1.2 I-II loop, written in purple throughout the figure, followed by the selected Ca_V_2.1 cytoplasmic domain. (B-F) Mid-level confocal images of tsA201 cells co-transfected with mCherry-tagged JPH3 and PM-associated Ca_V_2.1 N-terminus (B), I-II loop (C), II-III loop (D), III-IV loop (E), or C-terminus (F). For each construct, the vertically arranged panels display the distribution of the junctophilin (top), the cytoplasmic domain (middle), and the merged image (bottom). All Ca_V_2.1 cytoplasmic domains localized to the PM, with the N-terminus, I-II loop, and C-terminus also showing strong nuclear signals (B, C, F). Among the constructs, only the Ca_V_2.1 II-III loop exhibited a segmented fluorescence pattern that closely overlapped with JPH3, indicating strong colocalization (D). In contrast, the other constructs were uniformly distributed on the PM regardless of JPH3 localization (B, C, E, F). (G) Pearson’s colocalization coefficients, calculated from confocal sections at the cell’s bottom surface. Data are presented as individual cell values (circles), with the mean indicated by the height of the overlaid rectangles and ± SD by the error bars. Sample size is shown in parentheses as total number of cells analyzed/separately transfected dishes. (H) Mid-level confocal sections of a tsA205 cells transfected with either the PM-tethered Ca_V_2.1 II-III loop [(GFP-(I-II loop)-(Ca_V_2.1 II-III loop)] or plain II-III loop [GFP-(Ca_V_2.1 II-III loop)] without junctophilins. While GFP-tagged Ca_V_2.1 II-III loop shows a prominent reticular distribution, the PM-tethered loop clustered at the periphery of the cell in segmented areas that closely resemble those formed by full-length junctophilins. Scale bars = 5 µm.


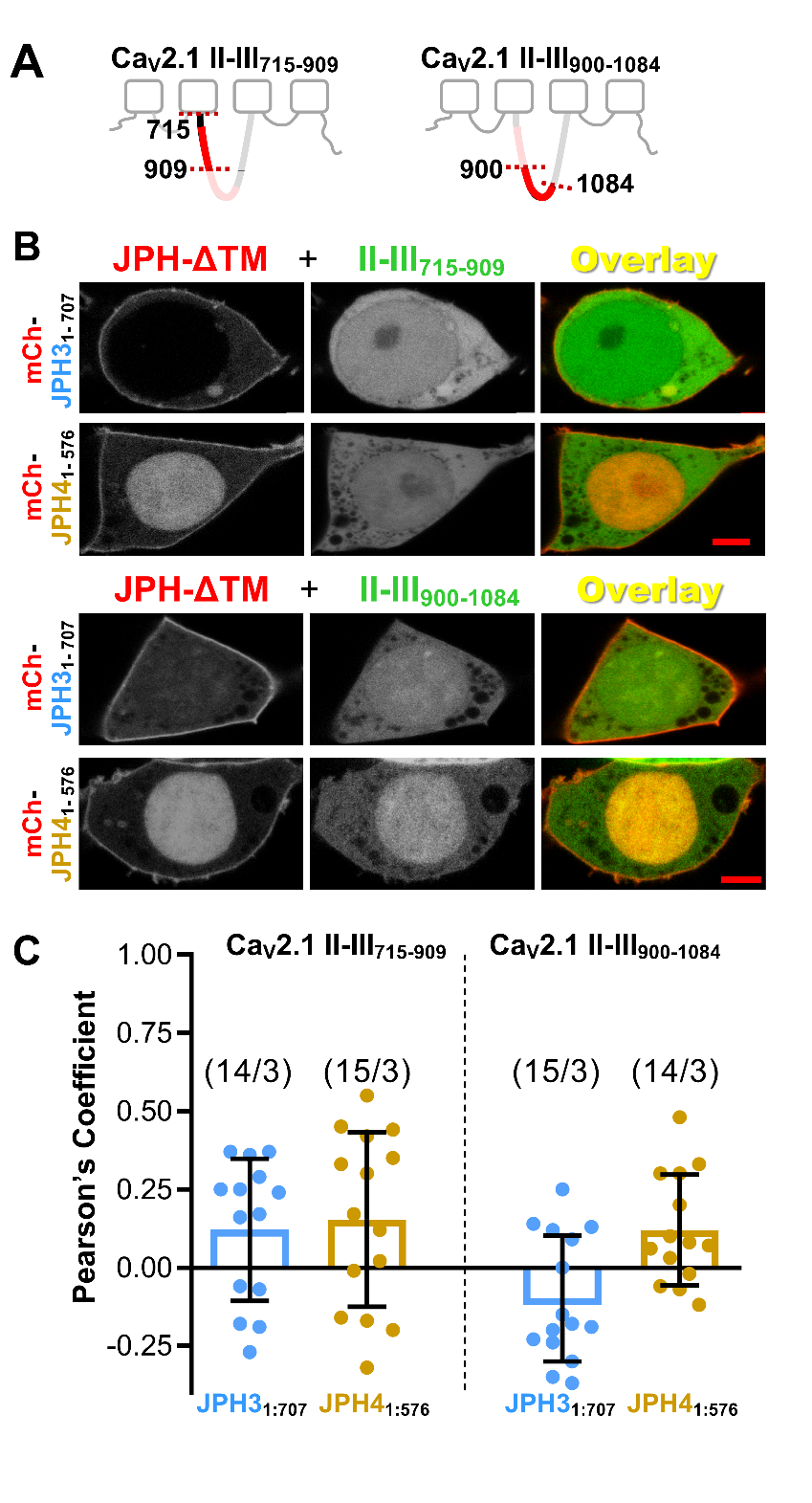
**Supplemental Figure 2**

**Supplemental Figure 2. The N- and C-terminal halves of the synprint domain do not interact with JPH3_1-707_ or JPH4_1-576_**. (A) Schematic representation of II-III_715-909_ and II-III_900-1084_, which contain the N-terminal or C-terminal halves, respectively, of the synprint domain (red) present in the II-III loop of Ca_V_2.1. (B) Midlevel optical sections of tsA201 cells expressing either mCherry-tagged JPH3_1-707_ or mCherry-JPH4_1-576_ in combination with either II-III_715-909_ or II-III_900-1084_ tagged with GFP. There was no apparent colocalization between II-III_715-909_ or II-III_900-1084_ and either JPH3 or JPH4. Bars = 5 µm. (C) Pearson's coefficients calculated from midlevel confocal sections. Circles indicate values for individual cells, with the mean ± SD indicated by the superimposed rectangle and horizontal black lines, respectively. Numbers in parentheses represent total number of analyzed cells/separate transfected dishes.


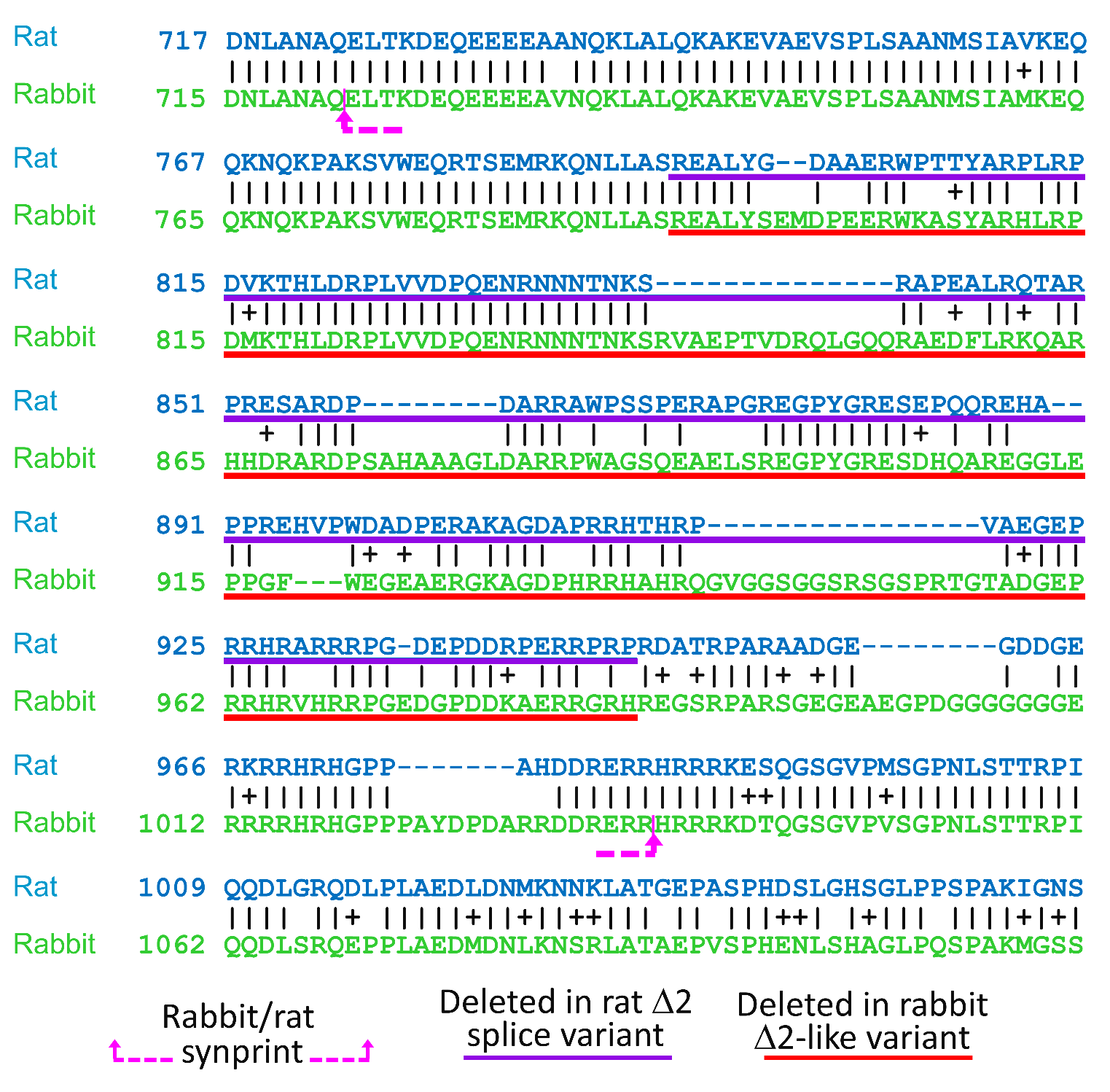
**Supplemental Figure 3**

**Supplemental Figure 3. Alignment of the synprint-containing segments of the Ca_V_2.1 II-III loops from rat and rabbit**. The illustrated sequences (accession numbers NM_012918 and 1709354B for rat and rabbit, respectively) begin at the first rabbit residue downstream of repeat II (18) and were aligned with SnapGene^TM^ using Blosum62. Introduced gaps are indicated by dashes, identical residues by vertical lines, and conserved residues by the plus sign. Residues 722-1036 which contain the synprint domain of rabbit Ca_V_2.1 (4), residues 793-948 which are deleted in the rat Δ2 splice variant (23) and the corresponding residues (791-985) that are deleted in the Δ2-like rabbit variant, are indicated by the pink arrows, purple underlining, and red underlining, respectively.
